# RNF8 enhances the sensitivity of PD-L1 inhibitor against melanoma through ubiquitination of galectin-3 in stroma

**DOI:** 10.1038/s41420-023-01500-3

**Published:** 2023-06-30

**Authors:** Yanan Guo, Rong Shen, Keren Yang, Yutong Wang, Haoyun Song, Xiangwen Liu, Xin Cheng, Rile Wu, Yanfeng Song, Degui Wang

**Affiliations:** 1 School of Basic Medical Sciences, Lanzhou University, Lanzhou, Gansu 73000 China; 2 NHC Key Laboratory of diagnosis and therapy of Gastrointestinal Tumor, Lanzhou, 730000 China

**Keywords:** Immune evasion, Melanoma

## Abstract

The failure of melanoma immunotherapy can be mediated by immunosuppression in the tumor microenvironment (TME), and insufficient activation of effector T cells against the tumor. Here, we show that inhibition of galectin-3 (gal-3) enhances the infiltration of T cells in TME and improves the sensitivity of anti-PD-L1 therapy. We identify that RNF8 downregulated the expression of gal-3 by K48-polyubiquitination and promoted gal-3 degradation via the ubiquitin–proteasome system. RNF8 deficiency in the host but sufficiency in implanted melanoma results in immune exclusion and tumor progression due to the upregulation of gal-3. Upregulation of gal-3 decreased the immune cell infiltration by restricting IL-12 and IFN-γ. Inhibition of gal-3 reverses immunosuppression and induces immune cell infiltration in the tumor microenvironment. Moreover, gal-3 inhibitor treatment can increase the sensitivity of PD-L1 inhibitors via increasing immune cell infiltration and enhancing immune response in tumors. This study reveals a previously unrecognized immunoregulation function of RNF8 and provides a promising strategy for the therapy of “cold” tumors. Tremendous effects of melanoma treatment can be achieved by facilitating immune cell infiltration combined with anti-PD-L1 treatment.

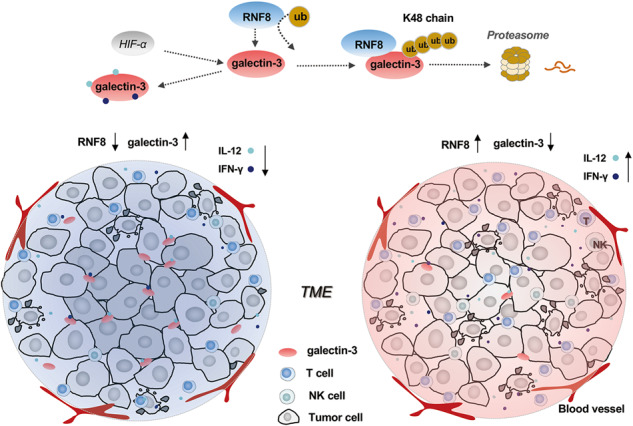

## Introduction

Targeted PD-1/PD-L1 therapy is an effective treatment for melanoma [1]. PD-1/PD-L1 blocking antibodies serve as key factors in TME, by re-invigorating exhausted T cells and thereby reviving pre-existing anti-tumor immunity [2]. However, more than half of the patients failed to achieve the therapeutic effect, due to the extremely complicated internal environment and variable objective response rate. Researches on the failure of elimination of tumor suggest that it is mainly because of the insufficient immune response against tumor [3]. Thus, strategies to enhance immune response and remove immunosuppression have been considered as the central goal to improve the therapeutic effect.

TME is characterized as the multiple complex components and signal crosstalk, which is composed of multiple cells and extracellular matrix (ECM) providing structural support for tumor cells, energy source, and signal transduction. In solid tumors, insufficient infiltration of cytokines and immune cells restricted by ECM components results in poor immune response and tumor immune escape, which is closely related to the immunotherapy effect [4].

Gal-3 presents a high binding affinity for β-galactosidase and glycosylated molecules. Studies have shown that gal-3 is increased in various tumors, and its upregulation in TME is closely related to immune response and tumor progression [5]. Emerging evidence suggests that TME may induce gal-3 expression to keep cellular homeostasis and promote cell survival [6]. Gal-3 could suppress activation of cytotoxic T cells, and shield ligands on the surface of tumor cells, thus leading to immunosuppression [7]. Gal-3 has been considered to be a potential target for clinical therapy since blocking gal-3 could improve the efficiency of tumor treatment [8]. Nevertheless, the underlying mechanisms that gal-3 regulates immunosuppression in TME remain not substantially elucidated.

RNF8 is a ubiquitin E3 ligase expressed ubiquitously in multiple tissues. Some previous works have improved that RNF8 plays essential roles in histone ubiquitination in DNA damage response [9]. RNF8 affects DNA damage response by inducing G2/M checkpoint activation [10], and downregulation of the G2/M checkpoint gene was significantly associated with prolonging progression-free survival with immune checkpoint inhibitors therapy and aided in the treatment of melanoma [11]. On the one hand, RNF8 mediated Akt activity to influence chemoresistance in lung cancer cells [12] and promoted triple-negative breast cancer progression via modifying Twist protein [8]. On the other hand, ARID1A regulated RNF8-mediated Chk2 ubiquitination, which improved cancer cell–intrinsic innate immunity to enhance the antitumor activity of immune checkpoint blockade [13]. Furthermore, RNF8 is also involved in many other biological processes, such as spermatogenesis [5], neurodegeneration [14], and epithelial–mesenchymal transition [15].

In this study, we found that RNF8 plays an essential role in maintaining the stability of TME. We demonstrated that depletion of RNF8 in vivo induced gal-3 expression and suppressed immune cell infiltration as well as the cytokines recruitment in TME, leading to an increase the tumor progression compared with RNF8 wildtype (RNF8^+/+^) mice. Gal-3 inhibited immune cell infiltration in TME through binding with cytokines IL-12 and IFN-γ, and the internal environment of tumor regulated gal-3 expression and distribution. Moreover, RNF8 regulated gal-3 K48 linked polyubiquitination to promote gal-3 degradation via the ubiquitin–proteasome system. Gal-3 intervention collaborated with PD-L1 inhibition and improved the antitumor effect significantly, which provided a promising therapeutic strategy against tumors by converting immune-excluded TME into immune-infiltrated TME.

## Results

### RNF8 deficiency in stroma accelerated the progression of melanoma and suppressed immune cells infiltration in TME

**Fig. 1:**
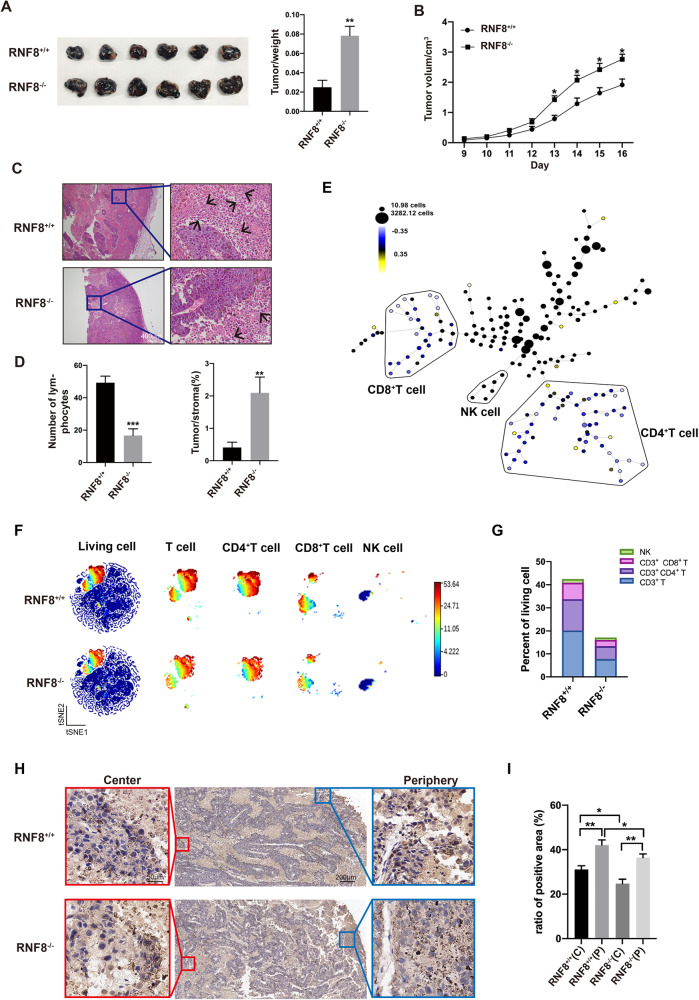
RNF8 deficiency in stroma accelerated the progression of melanoma and suppressed immune cell infiltration in TME. The 3-month-old RNF8^+/+^ and RNF8^−/−^ male mice were implanted by subcutaneous injection with 5 × 10^6^ B16F10 cells into the flank skin. **A** The tumor-beared RNF8^+/+^ and RNF8^−/−^ mice were compared (left), and the ratio of tumor weight was analyzed (right). **B** Volume of melanoma in RNF8^+/+^ and RNF8^−/−^ mice. **C** HE staining of the melanomas in groups. The arrow points to the lymphocytes. **D** The number of lymphocytes in groups (left), and the ratio of tumor area compared to stroma area (right). **E** SPADE analysis of tumor-infiltrating lymphocyte (TIL) in tumors from RNF8^−/−^ and RNF8^+/+^ hosts, and the TIL in RNF8^+/+^ was as a baseline and shown in black. Blue indicates a decrease in cell number from baseline. **F** t-SNE plot of TILs including CD3^+^ T cells, CD4^+^ T cells, CD8^+^ T cells, and NK cells. It represents a gradual increase in the number of cells from blue to red. **G** CyTOF analysis and frequencies of total CD3^+^ T cells, CD3^+^ CD4^+^ T cells, CD3^+^ CD8^+^ T cells, and NK cells. **H**, **I** Immunohistochemical staining of CD3 in the tumor, the tumor center area (center, C) and tumor periphery area (periphery, P) were amplified and analyzed. The values were presented as the mean ± SD (*n* = 3). Student’s *t*-test; *P < 0.05, **P < 0.01, ***P < 0.001.

To investigate the effect of RNF8 deficiency in tumor stroma on TME, RNF8^+/+^ and RNF8^−/−^ mice were inoculated with melanoma B16F10 cells respectively. Knockout of RNF8 in host mice significantly accelerated the progression of melanoma (Fig. 1A, B). HE staining showed that fewer lymphocyte cells were observed in the TME of RNF8^−/−^ mice (Fig. 1C, D). S100 and HMB45 positive cells in melanoma were enhanced in RNF8^−/−^ mice (Supplementary Fig. 1A, B), which implied that RNF8 deficiency in TME promoted melanoma progression.

Subsequently, we detected the immune cell infiltration of melanoma in RNF8^+/+^ and RNF8^−/−^ mice by mass cytometry (CyTOF). SPADE analysis distinctly identified the variant immune cell populations in TME between RNF8^+/+^ and RNF8^−/−^ group (Fig. 1E). In-depth analysis of immune cells by t-SNE identified CD3^+^ T cells, CD3^+^ CD4^+^ T cells, CD3^+^ CD8^+^ T cells and NK cells clusters, moreover, these clusters were decreased in melanoma-beared RNF8^−/−^ mice (Fig. 1F, G). For investigating differential pattern of immunity in melanoma, we defined 75 percent in central area as the tumor center, and the rest as tumor periphery (Supplementary Fig. 1C). Results revealed that CD3 expression was significantly higher in tumor periphery than center, moreover, CD3 expression in both tumor center and periphery from RNF8^−/−^ mice was more obvious lower than from RNF8^+/+^ mice (Fig. 1H, I, Supplementary Fig. 1D, E).

### RNF8 mediated infiltration of T cells in TME through IL-12/IFN-γ axis

**Fig. 2:**
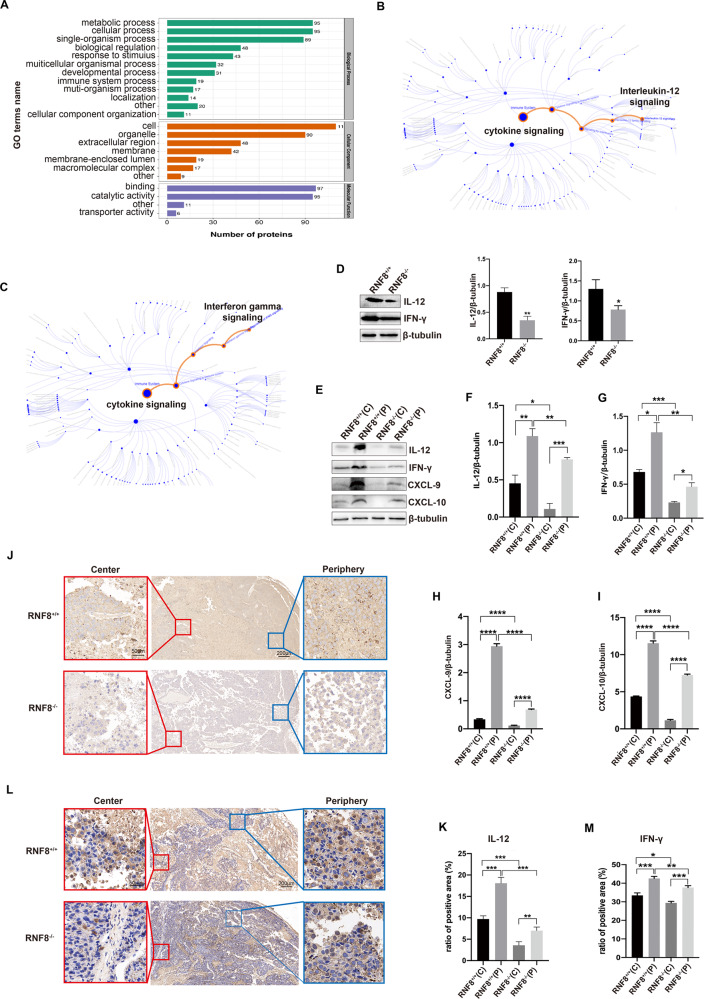
RNF8 mediated the infiltration of T cells in TME through IL-12/IFN-γ axis. **A** Gene Ontology (GO) analysis between shRNF8 and shCtrl cells. **B** The pathway enrichment analysis of IL-12 signaling. **C** The pathway enrichment analysis of IFN-γ signaling. **D** Western blot of IL-12 and IFN-γ expression in tumor. **E**–**I** Western of IL-12, IFN-γ, CXCL-9, and CXCL-10 in tumor center (C) and periphery (P) beared in RNF8^+/+^ and RNF8^−/−^ host. **J**–**M** Immunohistochemical staining of IL-12 (J) and IFN-γ (L) in the tumor. The analysis of positive ratio of IL-12 (**K**) and IFN-γ (**M**) in groups. All values were presented as the mean ± SD (*n* = 3). Student’s *t*-test; *P < 0.05, **P < 0.01, ***P < 0.001, ****P < 0.0001.

By post-translational modifications omics of ubiquitination, we found that immune response of related signal molecules was abnormal remarkably, in which the cytokines, IL-12 and IFN-γ, caused attention due to their immune regulation effect (Fig. 2A–C). Studies have indicated that IL-12 and IL-2 could activate T cells and NK cells to promote IFN-γ generation by helper T-cells [16]. The IL-12 and IFN-γ expression in melanoma from RNF8^−/−^ mice were reduced (Fig. 2D). Furthermore, we investigated expression difference of IL-12 and IFN-γ in tumor center and periphery. Surprisingly, expression of IL-12, IFN-γ, CXCL-9, and CXCL-10 in tumor center was dramatically decreased than tumor periphery (Fig. 2E–I). Consistently, IHC staining of IL-12 and IFN-γ in tumor got the similar results (Fig. 2J–M). The remarkable distribution difference was more typical in RNF8^−/−^ mice, cytokines in tumor center performed remarkably decreased. The specific distribution pattern of cytokines exactly conformed with the infiltration of T cells in TME. Therefore, RNF8 could mediate T cells infiltration by regulating IL-12/IFN-γ axis expression in TME.

### The distribution and content of IL-12 and IFN-γ was regulated by gal-3 in TME

**Fig. 3:**
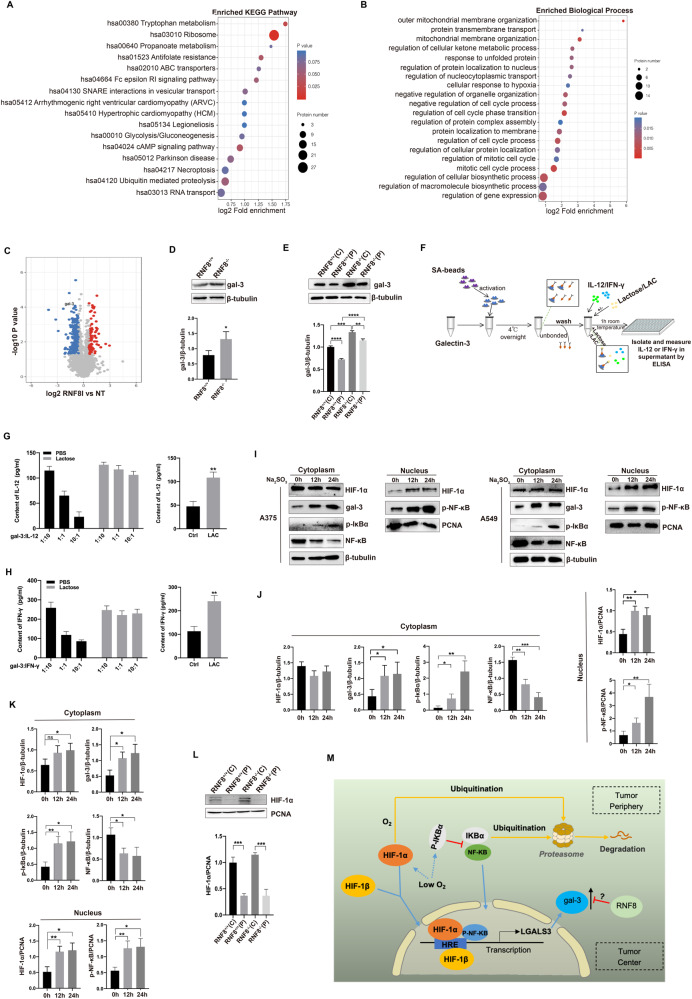
RNF8 deficiency results in gal-3 upregulation. **A** Gene set enrichment analysis showing changed pathways in RNF8 deficieny cells. **B** Gene set enrichment analysis showing changed biological process in RNF8 deficieny cells. **C** Volcano plot of ubiquitination modifications omics in RNF8 deficiency cells. **D** Western blot of gal-3 in tumor-beared RNF8^+/+^ and RNF8^−/−^ mice. **E** Western blot of gal-3 in center and periphery of tumor-beared RNF8^+/+^ and RNF8^−/−^ mice. **F** The technical routine of gal-3 binding with IL-12 or IFN-γ. **G** IL-12 and (**H**) IFN-γ content in the supernatant were measured by ELISA after incubation with gal-3-coated beads in the presence or absence of 100 mM lactose (left) or 50 mM LAC (right). The human melanoma A375 cells and human lung cancer A549 cells were treated with 0.2 g/L Na_2_SO_3_. **I**–**K** Gal-3, HIF-1α, p-IκBα, NF-κB, and p-NF-κB expression in A375 and A549 were detected by western blot at different time after treating with Na_2_SO_3_. **L** Western blot investigation of HIF-1α in tumor center and periphery in RNF8^+/+^ and RNF8^−/−^ mice. **M** The schematic diagram of a mechanism related to gal-3 in TME. All values were presented as the mean ± SD (*n* = 3). Student’s *t*-test; *P < 0.05, **P < 0.01, ***P < 0.001, ****P < 0.0001.

Cytokines are identified as the key mediators of intercellular communication and immune activation in TME, which play important roles in immune response and anti-tumor. The expression dysregulation of cytokines has been indicated in all the tumor types that have been detected. Based on the remarkable distribution and expression difference of IL-12 and IFN-γ in melanoma particular in RNF8^−/−^ mice, we investigated ubiquitination modifications omics data of control and RNF8-knockdown cells (Fig. 3A–C). Analysis revealed that RNF8 deficiency related to immunity, ubi-conjugation pathway and intracellular signal transduction. Among these genes, gal-3, a lectin that involved in binding with various proteins, was chosen for further investigation. Western blot detection showed that tumors from RNF8^−/−^ mice showed more gal-3 expression than from RNF8^+/+^ mice (Fig. 3D). Moreover, Gal-3 was highly expressed in tumor center than periphery (Fig. 3E). Gal-3 has been reported to increase in multiple tumors and bind with glycoproteins by forming lattices, such as cytokines which exist in glycosylated form mostly in tumor [17]. Then we tested the interaction of gal-3 with cytokines IL-12 and IFN-γ. The dynabeads were incubated with recombinant human gal-3, then IL-12 and IFN-γ were incubated with gal-3-coated beads respectively with lactose or LacNAc (LAC), the content of IL-12 and IFN-γ in solution was detected by ELISA for evaluating the capture status (Fig. 3F). Results indicated that IL-12 and IFN-γ were bound by gal-3 in a dose-dependent manner, and were released under the condition of lactose or LAC presence due to competitive binding (Fig. 3G, H).

Next, we investigated the gal-3 generation in tumor. Hypoxia could enhance transcription of gal-3 [18]. Due to the rapid growth of tumors, blood vessels formation near tumor center is commonly deficient, leading to insufficient energy supply of ischemia and hypoxia, and increasing hypoxia inducible factor-1α (HIF-1α) expression. Na_2_SO_3_ was used for establishing hypoxic cell model. After intervention for 0, 12 and 24 h in A375 and A549 cells, HIF-1α in cytoplasm and nucleus was detected. Results indicated that HIF-1α nuclear translocation was elevated in a time-dependent manner, consist with the conclusion that HIF-1α regulates gene transcription via nuclear translocation from cytoplasm [19]. NF-κB is another important regulator of gal-3 contributed to tumor growth and metastasis [20]. Our results demonstrated that phosphor-nuclear factor kappa B (p-NF-κB) was increased in nucleus with time, meanwhile, Phospho-Inhibitory Subunit of NF Kappa B Alph (p-IκBα) in cytoplasm was enhanced (Fig. 3I–K). The expression of HIF-1α in tumor center was increased compared with tumor periphery, however, the expression of HIF-1α has no significant change in tumor-beared RNF8^+/+^ and RNF8^−/−^ mice (Fig. 3L). Collectively, the different distribution of gal-3 in tumor was caused by multiple factors (Fig. 3M). These results suggest that the expression of gal-3 is mainly regulated by RNF8, thus led to the unbalance of cytokines and immune cells in TME.

### RNF8 interacted with gal-3

**Fig. 4:**
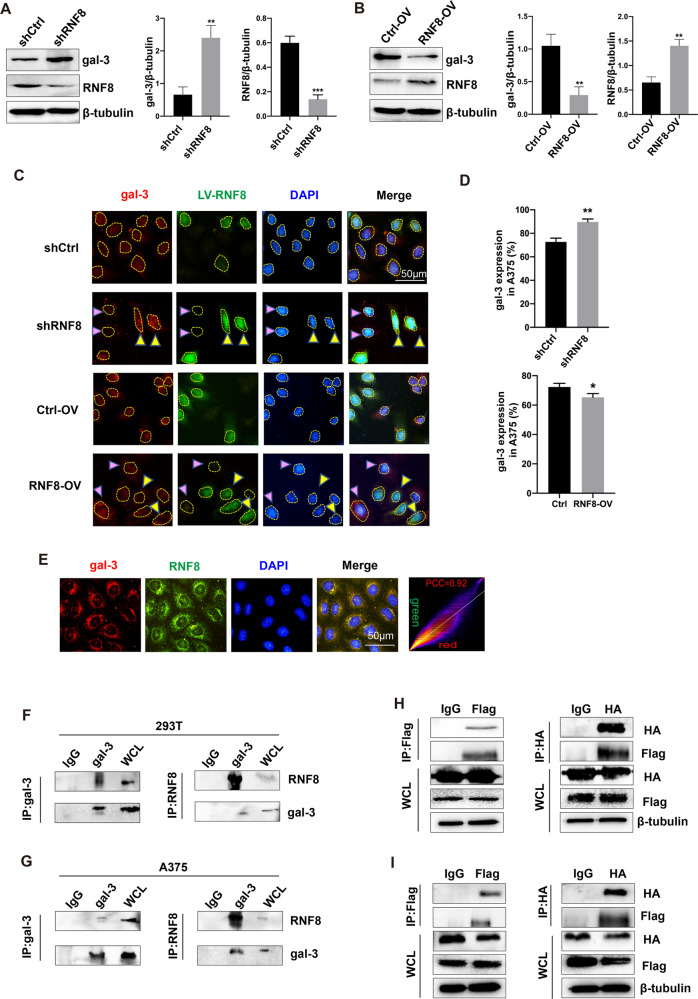
RNF8 interacted with gal-3. **A** Western blot investigation of RNF8 and gal-3 expression in shCtrl and shRNF8 groups in 293 T cell. **B** RNF8 and gal-3 expression in Ctrl-OV and RNF8-overexpression (RNF8-OV) groups. **C** A375 cells were transfected with LV-RNF8 for shRNF8 or RNF8-OV. The yellow arrows indicated the successful transfected cells, and the purple arrows indicated the non-transfected cells. **D** The statistical analysis of gal-3 expression in A375 cells. **E** Fluorescent staining of endogenous RNF8 and gal-3 distribution in A375 cells. **F**, **G** Co-IP analysis of interaction between endogenous RNF8 and gal-3 in 293 T (**F**) and A375 cells (**G**). **H**, **I** Co-IP analysis of interaction between exogenous RNF8 and gal-3 in 293 T (**H**) and A375 cells (**I**), wcl whole cell lysate. The values were presented as the mean ± SD (*n* = 3). Student’s *t*-test; *P < 0.05, **P < 0.01, ***P < 0.001.

As shown in Fig. 4A, a remarkable enhancement of gal-3 was observed after RNF8 knockdown. In contrast, RNF8 over-expression led to a depression of gal-3 (Fig. 4B). The result was confirmed by immunofluorescent counterstaining of RNF8 and gal-3 (Fig. 4C, D). And according to the human protein ATLAS data (Supplementary Fig. 2A), we screened out skin with high level of galectin-3 and liver with low expression. WB results showed that the expression of galectin-3 in the liver and skin from RNF8-deficient mice was both higher than that from RNF8^+/+^ mice (Supplementary Fig. 2B, C), which was consistent with cells results. In addition, immunofluorescent staining showed that RNF8 and gal-3 was co-localized in cytoplasm (Fig. 4E), indicating the potential links between RNF8 and gal-3. An interaction between endogenous RNF8 and gal-3 was observed (Fig. 4F, G), this was confirmed by interaction between Flag-RNF8 and HA-gal-3 over-expressed in both 293 T and A375 cells (Fig. 4H, I). These data clearly suggested that gal-3 interacted with RNF8 and was negatively regulated by RNF8.

### RNF8 mediated degradation of gal-3 via promoting K48-linked ubiquitination

**Fig. 5:**
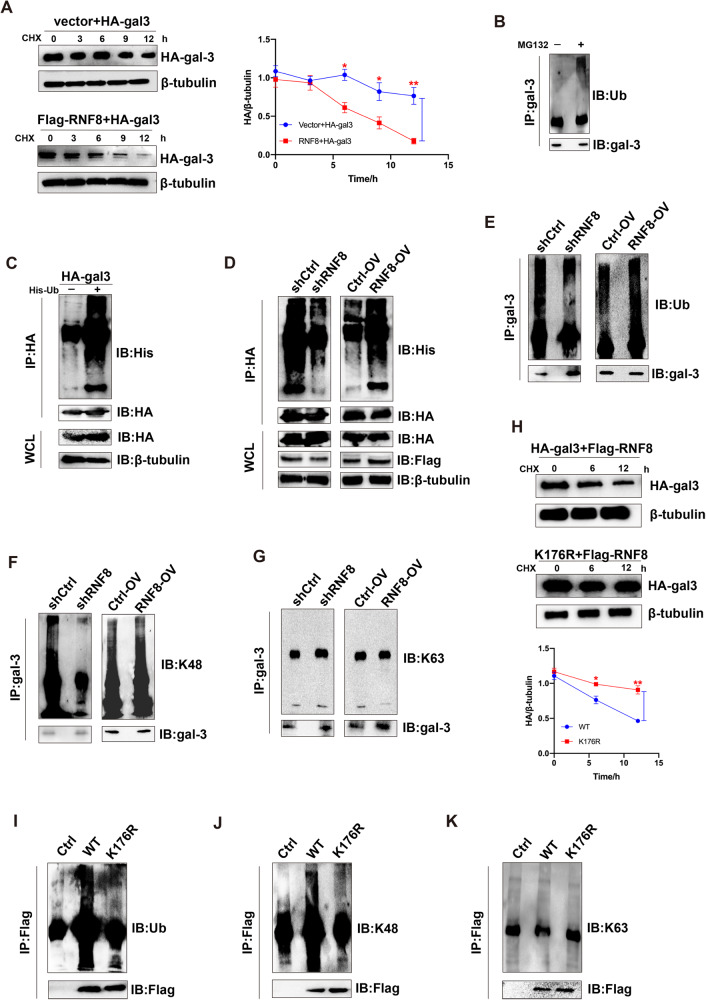
RNF8 mediated K48-linked polyubiquitination and degradation of gal-3 through ubiquitin–proteasome system. **A** CHX (5 μg/mL) was used in 293 T cells to measure gal-3 degradation. **B** 293 T cells were treated with or without 10 mmol/L MG132 for 4 h. The ubiquitination level of gal-3 was detected. **C** HA-tagged gal-3 and His-tagged Ub plasmids were co-transfected into 293 T cells for 36 h, followed by cell lysate preparation and IP assay with anti-HA beads followed by immunoblotting with indicated antibodies. **D** 293 T cells were treated with exogenous RNF8 (shRNF8 or RNF8-OV), gal-3 and Ub, and the ubiquitination of HA was investigated. **E**–**G** 293 T cells were transfected with LV-shRNF8 or LV-RNF8-OV, and the ubiquitination (**E**), K48 (**F**), and K63 (**G**) of gal-3 were investigated. **H** 293 T cells were transfected with gal-3K176R (mutation of gal-3 at lysine 176 site), the expression of gal-3 was investigated. **I**–**K** 293 T cells were transfected with Flag-Ctrl, Flag-LGALS3, and Flag-K176R, and the total ubiquitination (**I**), K48 linked (**J**), and K63-linked (**K**) ubiquitination was detected.

The cycloheximide (CHX) chase assays indicated that RNF8 supplementary (Flag-RNF8) significantly enhanced gal-3 (HA-gal-3) degradation in a time-dependent manner (Fig. 5A). The proteasome inhibitor MG132 treatment partially enhanced endogenous ubiquitinated gal-3 in 293 T and A375 cells, respectively (Fig. 5B, Supplementary Fig. 3A). Consistently, ubiquitinated HA-gal-3 was increased obviously once supplemented exogenous ubiquitin (His-ub) in two cell lines (Fig. 5C, Supplementary Fig. 3B). Next, exogenous His-ub and HA-gal-3 were used to detect the level of ubiquitinated gal-3. The ubiquitinated exogenous gal-3 was reduced in RNF8-KD (Flag-shRNF8) cells but elevated after RNF8 over-expressed (Fig. 5D, Supplementary Fig. 3C). The whole ubiquitination level of gal-3 by endogenous ubiquitin revealed similar results (Fig. 5E, Supplementary Fig. 3D).

Then we compared the K48 and K63 ubiquitination level of gal-3 in RNF8-KD and RNF8 over-expressed cells. The K48-ubiquitinated gal-3 was significantly reduced in RNF8-KD cells compared with control, and RNF8 overexpression enhanced the K48 polyubiquitination of gal-3 (Fig. 5F, Supplementary Fig. 3E). However, the K63-ubiquitination of gal-3 showed no response to the intervention (Fig. 5G, Supplementary Fig. 3F). To explore the necessary residue required for gal-3 ubiquitination, we constructed the gal-3 mutant according to LC–MS/MS analysis of purified gal-3 protein (Supplementary Fig. 3G), in which lysine residue was replaced with arginine residue. The cycloheximide chase assays performed that K176R significantly reversed HA degradation in 293 T cells (Fig. 5H). As expected, K176 mutation abolished the K48-ubiquitination of gal-3 in both 293 T and A375 cells (Fig. 5I–K, Supplementary Fig. 3H, I). Collectively, RNF8 regulated gal-3 K48 linked polyubiquitination to promote gal-3 degradation via ubiquitin–proteasome system.

### Intervention of gal-3 remolded the tumor immune microenvironment

**Fig. 6:**
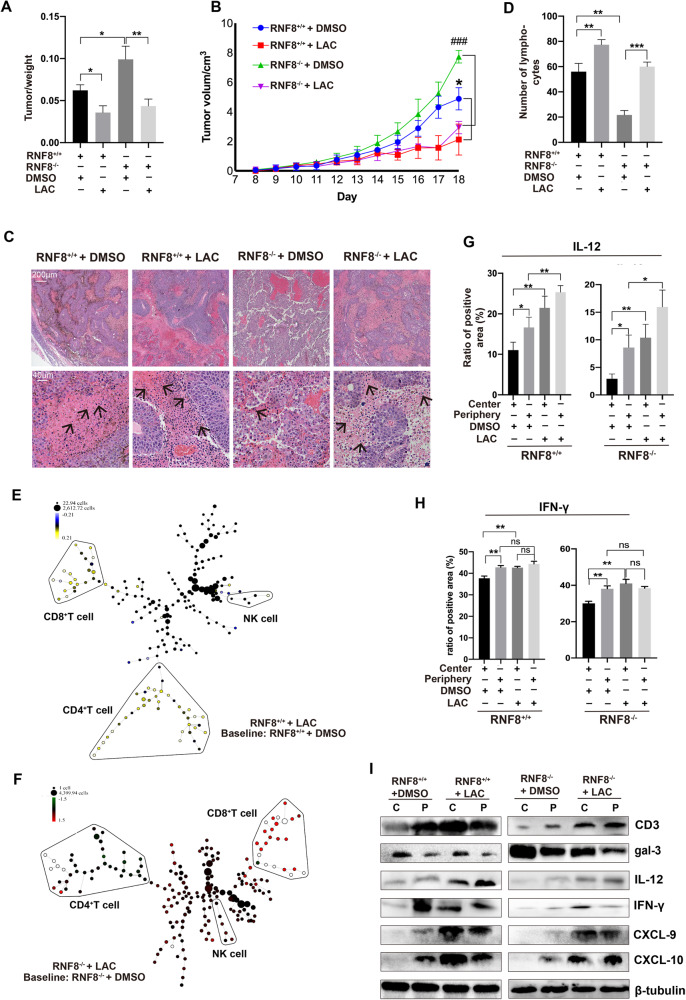
The infiltration of cytokines and immune cells were enhanced by inhibiting gal-3 in TME. **A**, **B** LAC was injected intratumorally at a single dose of 0.1 μmol per mice after tumor formation, the ratio of tumor weight (**A**), tumor volumes (**B**) were detected. **C**, **D** HE staining of implanted melanomas in RNF8^+/+^ and RNF8^−/−^ mice treated with DMSO or LAC. The arrow points to TILs. **E**, **F** SPADE analysis of TILs in RNF8^+/+^ + DMSO, RNF8^+/+^ + LAC, RNF8^−/−^ + DMSO, and RNF8^−/−^ + LAC mice. **G** Analysis of IL-12 by immunohistochemical staining (Supplementary Fig. 7A) of tumors in above groups. **H** Analysis of IFN-γ by immunohistochemical staining (Supplementary Fig. 7B) of tumors in above groups. **I** Western blot of CD3, gal-3, IL-12, IFN-γ, CXCL-9, and CXCL-10 expression in tumor center and periphery in above groups. The values were presented as the mean ± SD (*n* = 3). Student’s *t*-test; *P < 0.05, **P < 0.01, ***P < 0.001.

It has been reported that LacNAc (LAC) plays a significant affinity with gal-3 [21]. In this study, B16F10 cells were injected subcutaneously into RNF8^+/+^ and RNF8^−/−^ mice, and then LAC was injected into tumor center. Results showed that long-term treatment with LAC delayed melanoma growth and progression in both RNF8^+/+^ and RNF8^−/−^ groups (Fig. 6A, B). HE exhibited that LAC treatment improved immune cells infiltration in TME and suppressed tumor growth (Fig. 6C, D, Supplementary Fig. 4A). IHC staining of CD3^+^ T cells infiltration in melanoma was increased after LAC intervention, particularly in RNF8^−/−^ mice, which performed fewer immune cells infiltration in TME (Supplementary Fig. 4B, C).

Subsequently, immune cells subsets in tumor of RNF8^+/+^ and RNF8^−/−^ mice were investigated by CyTOF. The t-SNE analysis was presented for visualized observation of the immune cell populations changes of LAC treated melanoma in RNF8^+/+^ and RNF8^−/−^ group (Supplementary Fig. 4D). SPADE analysis presented the subsets ratio change in RNF8^+/+^ and RNF8^−/−^ mice treated with LAC, compared with that treated with DMSO (Fig. 6E, F). The CD3^+^CD4^+^, CD3^+^CD8^+^ and NK cells in tumor from RNF8^−/−^ mice were obviously lower than RNF8^+/+^ mice, LAC treatment significantly increased infiltration of CD3^+^CD4^+^, CD3^+^CD8^+^ and NK cells in tumors both in RNF8^+/+^ and RNF8^−/−^ mice (Supplementary Fig. 5). Consistently, CyTOF analysis performed that the pro-inflammatory cytokines IL-2 and IFN-γ expression was decreased, and anti-inflammatory cytokines IL-4 and IL-10 level was increased in RNF8^−/−^ mice compared with RNF8^+/+^ mice, interestingly, LAC treatment improved the infiltration of cytokines in RNF8^−/−^ mice (Supplementary Fig. 6).

IHC staining analysis demonstrated that IL-12 and IFN-γ positive cells in tumor center were increased in RNF8^+/+^ and RNF8^−/−^ mice after LAC treatment (Fig. 6G, H, Supplementary Fig. 7). Western blot presented that LAC intervention for a long-term partially reduced gal-3 expression in the tumor center and periphery. Surprisingly, the increase of CD3, IL-12, IFN-γ, CXCL-9, and CXCL-10 expression in LAC-treated tumors was much higher than in tumors treated with DMSO (Fig. 6I, Supplementary Fig. 8). These data suggest that inhibition of gal-3 could relieve immunosuppression through improving cytokines and immune cells infiltration even in the harsh TME.

### Inhibition of gal-3 enhanced sensitivity of PD-L1 inhibitor for melanoma

**Fig. 7:**
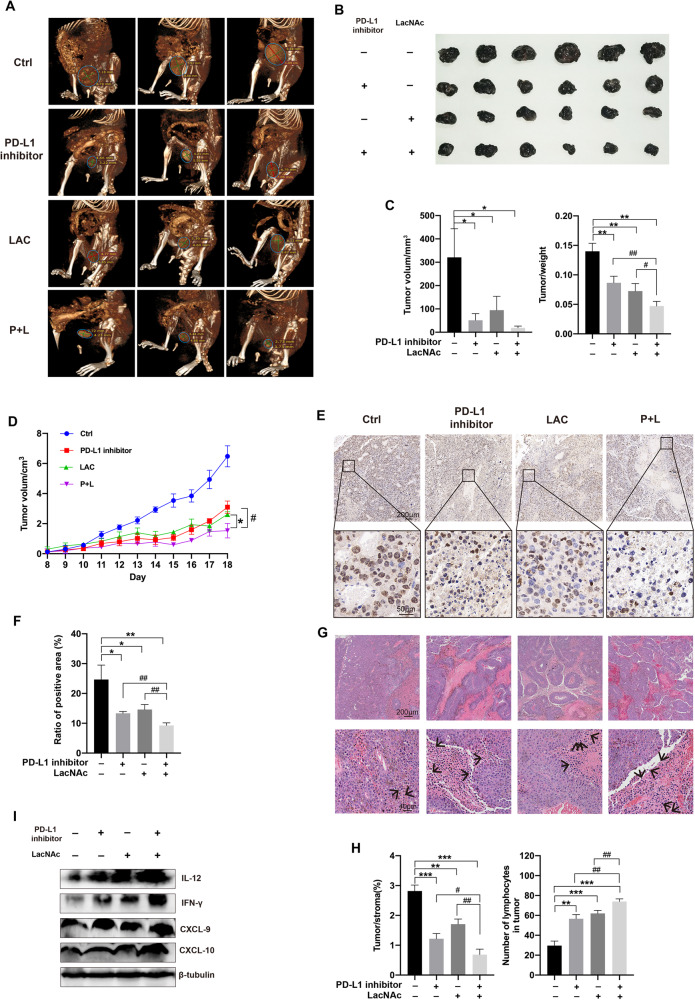
Inhibition of gal-3 enhanced sensitivity of PD-L1 therapy for melanoma. PD-L1 inhibitor was dosed by intraperitoneal injection at 1 μmol per mouse. LAC was dosed by intratumor injection at a single dose of 0.1 μmol per mouse. **A** The 3D modeling of subcutaneous tumor-beared in RNF8^+/+^ host that was treated with different strategies, including PD-L1 inhibitor, LAC, and PD-L1 inhibitor plus LAC (P + L). **B** The representative tumors in each group were shown. **C** The tumor volume (left) and the ratio of tumor weight (right). **D** Tumor growth volume in different groups. **E** The Ki-67 immunohistochemical staining of tumors. **F** Statistical analysis of Ki-67 in (**E**). **G** HE staining of melanomas in different groups. The arrow points to lymphocytes. **H** The ratio of tumor area compared to stroma area in each group (left). The number of lymphocytes in groups (right). **I** The western blot of IL-12, IFN-γ, CXCL-9, and CXCL-10 in PD-L1 inhibitor, LacNAc, and combination group. All values are presented as the mean ± SD (*n* = 6). Student’s *t*-test; *P < 0.05, **P < 0.01, ***P < 0.001.

The immune checkpoint blockade has verified the effectiveness of clinical treatment, however, due to poor immune infiltration, more than half of the patients fail to benefit from the therapy such as anti-PD-1, anti-LAG-3, and anti-CTLA-4. Based on the previous results, we treated melanoma with combined intervention including gal-3 inhibitor and PD-L1 inhibitor to examine the effect on immune remodeling and tumor progression. The results showed that combined therapy of PD-L1 inhibitor and LAC decreased tumor growth more dramatically than separately using PD-L1 inhibitor or LAC (Fig. 7A–D). IHC staining performed that the Ki-67 positive cells in the tumor were less in the combined therapy group compared with the monotherapy group (Fig. 7E, F). Consistently, HE analysis revealed that the infiltration of immune cells was increased in the combined therapy group (Fig. 7G, H). Western blot data also indicated that cytokines content in the tumor was remarkably increased after combined therapy compared with other groups, including IL-12, IFN-γ, CXCL-9, and CXCL-10 (Fig. 7I, Supplementary Fig. 9). Collectively, gal-3 inhibition combined with PD-L1 inhibition treatment performed a prominent efficacy of melanoma through enhancing immune infiltration and reducing immunosuppression in TME.

## Discussion

In respect of tumor recognition, response, and elimination, immune cells play a decisive role. These immune cells may process tumor-antagonizing or tumor-promoting functions. Although the tumor-antagonizing immune cells target and kill the tumor in the early stage of tumorigenesis, multiple cellular molecules have changed in TME to help tumor cells adapt to the microenvironment and maintain growth, which leads to tumor escape and treatment-resistant [22, 23]. Current studies mainly focus on remodeling TME, blockading immunosuppression, and enhancing immune cells’ response to improve the effect of tumor treatment. Recently, the immune checkpoint modulators (represented by anti-CTLA4 and anti-PD-1/PD-L1 antibodies) have presented unexpected antitumor effects [24]. This study focused on the TME component and indicated a new mechanism for regulating TME to prevent melanoma progression. We identified that RNF8-mediated ubiquitination was a crucial post-translational modification of gal-3. We investigated the regulation mechanism of immune exclusion by RNF8 mediated gal-3 ubiquitination via ubiquitin-proteasome system in TME, and the effect of gal-3 inhibition combined with immune checkpoint inhibitor treatment on melanoma.

Actually, most of the components in TME generate mainly from the host besides the tumor cells. And the components of TME are of importance in regulating immune escape and tumor progression. The current studies explore the effect of RNF8 on the progression and therapy resistance of tumor cells, based on the mechanisms related to DNA damage repair and genomic instability [25]. In this study, we demonstrated that RNF8 is an important regulator of gal-3, RNF8 deficiency of the host led to increased gal-3 level in TME and promoted implanted tumor progression (Figs. 1 and 3). Posttranslational modifications such as phosphorylation, ubiquitination, methylation, and proteolytic cleavage play an indispensable role in regulating the protein functions. Most studies related to gal-3 focused on its inhibition and proteolytic cleavage, few studies had been conducted on the ubiquitination of gal-3 and its function changes [26, 27]. A previous study showed that Calpain4 was critical to gal-3 tyrosine phosphorylation and ultimate secretion from the cell [28]. A recent study proposed that gal-3 could be efficiently cleaved by purified extracellular 20 S proteasome and thus disable its function [29]. Our results indicated that RNF8 interacted with gal-3, and that gal-3 was negatively regulated by RNF8 (Fig. 4). The ubiquitination assay in vitro confirmed that gal-3 was degraded through ubiquitin–proteasome system by RNF8 (Fig. 5, Supplementary Fig. 3). Furthermore, we found that knocking down RNF8 reduced ubiquitination level of gal-3, especially the K48-linked ubiquitination, but showed no effect on K63-linked ubiquitination (Fig. 5, Supplementary Fig. 3). Dramatically, mutation at the specific site of gal-3 K176 abolished the K48-linked ubiquitination, and also showed no effect on K63-linked ubiquitination. These results illustrated a clear regulatory relationship, RNF8 mediated gal-3 K48 linked polyubiquitination and promoted gal-3 degradation via ubiquitin–proteasome system.

The matrix remodeling enzymes, cytokines, and metabolites keep changing constantly and interact with each other, affecting tumor progression and therapy cooperatively [30]. Cytokines can be generated commonly by cells such as endothelial cells and fibroblasts for recruiting immune cells [31]. Nevertheless, components in TME could sequester cytokines, chemokines, and other molecules to contact immune cells and establish an immune excludes environment. An important regulatory element is glycoprotein gal-3, which has been suggested to influence immune cells and regulate their function negatively [32]. Gal-3 can be secreted and dispersed to the cell surface and ECM then binds with the glycosylated proteins to form lattices and restrains the diffusion of the glycosylated soluble factors in TME [33]. The restraint of cytokines by gal-3, such as IL-2 and IFN-γ, reduced downstream signal molecules production and function, thus resulting in immune exclusion in TME [34]. Moreover, the dense collagen deposition in TME has been proposed as an obstacle to the infiltration of immune cells into the tumor, the accumulation of gal-3 contributes to collagen deposition and thus intercepts immune cells’ entry into TME [35]. In this study, we demonstrated that RNF8 deficiency induced gal-3 high expression in TME, and accelerated melanoma growth with a deteriorated TME. In vitro experiment indicated that gal-3 could bind with IL-12 and IFN-γ in a dose-dependent manner, and lactose (LAC) intervention could competitively bind to gal-3, therefore, release the cytokines (Fig. 3). Consistently, RNF8 knockout of host resulted in significantly decreased IL-12 and IFN-γ in the tumor, which was directly related to gal-3 elevation (Fig. 2). Nevertheless, LAC injection in tumor effectively liberated IL-12 and IFN-γ and thus elevating downstream chemokines CXCL-9 and CXCL-10, particularly in RNF8 deficient mice (Fig. 6, Supplementary Figs. 6–8).

Several studies have suggested that IL-12-related cytokine therapy combined with immunotherapy presents an effective synergistic antitumor effect [36]. In clinical melanoma treatment, delivery of the IL-12 gene into the tumor elicited partially complete regression and tumor suppression in metastatic melanoma [37]. Recent studies also proposed that effective antitumor responses required sufficient IL-12 produced by dendritic cells upon sensing IFN-γ released by T cells, crosstalk between DC and T cells, to stimulate T cell immunity [38]. Our data confirmed that decreased pro-inflammatory cytokines (IL-2, IFN-γ, and IL-12) and elevated anti-inflammatory cytokines (IL-4, IL-10) in tumor-beared RNF8^−/−^ mice, yet inhibition gal-3 treatment effectively increased pro-inflammatory cytokines and decreased anti-inflammatory cytokines infiltration in TME (Supplementary Fig. 6). Furthermore, inhibition of gal-3 could release more IL-12 and IFN-γ in TME, which enhances the sensitivity of PD-L1 inhibitor treatment for melanoma and presents a more significant antitumor effect.

Gal-3 has been suggested to inhibit T cell activation and function through promoting TCR downregulation, and knockout of gal-3 increased TCR and IFN-γ expression in CD4^+^ T cells [39]. Studies indicated that gal-3 could bind with LAG-3 on CD8^+^ T cells and inhibit cell function [39]. Moreover, co-culture gal-3 deficient melanoma cells with CD8^+^ T cells significantly induced IFN-γ levels in CD8^+^ T cells, and promoted tumor-reactive T cell expansion [40]. In this study, we found that the infiltration of fewer immune cells in melanoma-beared RNF8 deficient host compared with the control host, including CD4^+^ T, CD8^+^ T, and NK cells (Fig. 2). Likewise, the intervention of gal-3 obviously increased immune cells infiltration in tumors in both RNF8^+/+^ and RNF8^−/−^ mice (Supplementary Fig. 5). These results suggested that RNF8 played a pivotal role for maintaining immune response in TME, through restricting gal-3 expression to ensure the cytokines and chemokines and immune cells infiltration.

Studies have indicated that the center and periphery of the tumor perform high epigenetic heterogeneity and transcriptional heterogeneity. It is widely known that hypoxia is a critical element to affect tumor diversity and progression, which is associated with energy metabolism, oxygen supply, and tumor angiogenesis [41]. Actually, hypoxia occurs in most solid tumor interiors during development because of the rapid growth and energy competition of tumor cells. Meanwhile, the development of hypoxic regions within rapidly growing tumors could also improve T-cell suppression via the increased production of adenosine in such areas [42]. It has been suggested that lower methylation in the tumor core is related to hypoxia, speculating that hypoxia in TME contributes to epigenetic changes and is related to the survival of patients [43]. Clearly, the heterogeneity in tumors will determine tumor adaptability and progression, and even affect tumor therapy effect [44]. We investigated the expression difference between the tumor center and periphery. Exactly, gal-3 expression in the tumor center was higher than that in the periphery especially in RNF8^−/−^ host (Fig. 3). As well, the distribution pattern of cytokines and chemokines in tumor performed site-dependent, low central expression and high peripheral expression (Fig. 2), which was opposite to gal-3 distribution. Then we detected a correlation between hypoxia and gal-3 expression by cell and mice models. The nuclear translocation of gal-3 regulators, HIF-1α and p-NF-κB, was increased in a time-dependent manner under the condition of hypoxia. What is more, the HIF-1α level in the tumor center was increased compared with the tumor periphery, yet it was no significant difference between RNF8^+/+^ and RNF8^−/−^ mice (Fig. 3). It suggests that the high expression of gal-3 in the tumor center and periphery from RNF8^−/−^ mice is related with the degradation of gal-3 via the ubiquitin–proteasome system.

By exploring the RNF8/gal-3/cytokine axis action in TME, we acquired a better understanding of tumor immune exclusion and designed an effective combination therapeutic strategy for melanoma. We treated melanoma with gal-3 inhibitor and PD-L1 inhibitor, which exhibited a superior effect than separate treatment in suppression of tumor development (Fig. 7, Supplementary Fig. 9), consistent with the previous studies on tumor therapy by inhibiting gal-3 and immune checkpoint synchronously [45]. In this study, combination therapy recovered IL-12/IFN-γ positive feedback mechanisms and induced immune cell infiltration in TME, through removing restriction of cytokines by suppressing gal-3, meanwhile, shutting down the PD-L1 function of tumor cells by inhibition of immune checkpoint. This strategy promoted remodeling of the immune microenvironment and enhanced immune response in melanoma and effectively inhibited tumor progression. Collectively, these results strongly indicated the effectiveness of this “cocktail strategy”, which provided a potential platform for the therapy of “cold” tumors.

## Conclusions

In summary, the present study indicated an essential regulatory role of RNF8-mediated ubiquitination degradation of gal-3 in maintaining immune response in TME. Due to the crucial effect of gal-3 on the restriction of cytokines and subsequent immune infiltration, we propose that targeting gal-3 and combining it with immune checkpoint inhibition may offer a potential strategy for melanoma immunotherapy.

## Materials and methods

### Cells lines and cells culture

A375 (CL-0014, Pricella), A549 (CL-0016, Pricella), B16F10 (CL-0319, Pricella), and 293 T cells (CL-0005, Pricella) were obtained directly from Procell, Wuhan, China. A375 and 293 T cells were cultured at 37 °C with 5% CO_2_ in complete DMEM medium (HyClone, SH30022.01) supplemented with 10% FBS (Gibco, 10100147C) and 1% penicillin/streptomycin (Solarbio, P1400). A549 and B16F10 cells were cultured at 37 °C with 5% CO_2_ in a complete 1640 medium (Cytiva, SH30809.01) supplemented with 10% FBS and 1% penicillin/streptomycin.

### Animals

The 3-month-old RNF8^−/−^ and RNF8^+/+^ male mice were used in our studies. The RNF8^−/−^ mice were provided by Xiaochun Yu as described previously [46]. The gene-deficient embryonic stem (ES) cell clone RRR260 was used to produce the RNF8^−/−^ mice. We used RNF8^+/-^ mice to produce RNF8^−/−^ mice. All animals were maintained in the SPF laboratory at the Lanzhou University Animal Center. The animals were fed under standard housing conditions and given free access to food and water. The experimental protocols were approved by the Committee on the Ethics of Animal Experiments of Lanzhou University.

### RNA and plasmids interference

Lentiviral vectors encoding human RNF8 and scrambled control shRNA were obtained from Genechem company. The specific sequences of LV-RNF8-RNAi were shown in Table S1 of supplementary materials. We transfected the A375 and 293 T cells by using hitransG P (GeneChem Co., Shanghai, China). The cDNA sequences of LGALS3 and UBB gene were delivered into GV362-CMV × 3(Flag, HA or HIS)-Neo and GV658-CMV × polyA-puro vector for conditional expression (GeneChem Co., Shanghai, China). The mutant construct of LGALS3 (K176R) was generated by site-directed mutagenesis and cloned into a GV362-CMV× 3Flag-Neo vector (GeneChem Co., Shanghai, China). Lipofectamine 3000 regents (Invitrogen, L3000001) was used for transient transfection according to the manufacturer’s instructions. The transfection efficiency was detected by fluorescent microscopy after 48 h.

### Mouse xenograft tumor models

The 3-month-old RNF8^−/−^ and RNF8^+/+^ male mice were used to build a tumor model. Then, 5 × 10^6^ B16F10 cells were subcutaneously injected into the flank of RNF8^+/+^ and RNF8^−/−^ mice respectively. Tumor sizes were recorded every day from palpating the tumor. LacNAc (FuShen, FS0234), the inhibitor of gal-3 was injected into the tumor center at a concentration of 0.1 μmol per tumor from Day 7 and injected every two days. PD-L1 inhibitor (AbMole, PD-1/PD-L1 inhibitor 1, M8959) was injected into the abdomen at a concentration of 1 μmol per mouse after 1 week. And 1 week later, the mice accepted repeatedly PD-L1 inhibitor therapy. Animals were sacrificed and subcutaneous tumors were harvested at the end of experiments.

### Mass cytometry

For CyTOF experiments, the mouse intracellular cytokine I panel kit was purchased from Fluidigm, and the CyTOF antibodies in the mouse lymphocytes phenotyping panel were purchased pre-conjugated directly from the supplier (PLTTECH, China). And the CyTOF antibodies and intracellular cytokine panel were listed in Table S2 and Table S3. TILs in melanoma were isolated using a mouse tumor-infiltrating tissue lymphocyte separation medium (Solarbio, P9000). After passing through a 100 μm filter membrane, the cells were washed twice with PBS and prepared for staining. Totally, 3 × 10^6^ cells were prepared for staining and testing. EQ four-element calibration beads (Fluidigm) was used to normalize the signal intensity according to the manufacturer’s instructions. The files were normalized and analyzed using the Cytobank platform on the website to gate the cell populations and handle high dimensional data.

### t-distributed stochastic neighbor embedding (t-SNE) analysis

A random subset of 10^4^ cells was selected for each sample and incorporated into a single expression matrix prior to t-SNE analysis. In addition to protein markers in t-SNE analysis, beads, event length, intercalator, viability, center, offset, residual, and time channel were removed from the expression matrix. A total of 10^5^ cells and 8 markers (Table S2) were used to create a t-SNE map of the TILs.

### Spanning tree progression of density normalized events (SPADE) analysis

Our data types were stored as flow cytometry standard (FCS) files, compatible with many analysis packages, and were represented as a data matrix with rows corresponding to individual cells and columns corresponding to markers of interest (CD3^+^ CD4^+^, CD3^+^ CD8^+^, NK). The generation of optimal SPADE input data required the selection of live and singlet events or cells, using DNA gates and event length basing constraints for large-scale cell assays or some form of cell viability probe.

### Antibodies

The following antibodies were used: RNF8 (Proteintech, # 14112-1-AP), β-tubulin (Affinity, # T0023), CD3 (Affinity, # DF6594), IL-12 (Affinity, # AF5133), IFN-γ (Affinity, # DF6045), CXCL-9 (Affinity, # DF9920), CXCL-10 (Affinity, # DF6417), galectin-3 (Proteintech, # 14979-1-AP), HIF-1α (Proteintech, # 66730-1-Ig), p-IκBα (Abcam, # ab133462), NF-κB (Abcam, # ab16502), P-NF-κB (Santacruze, # sc-101752), PCNA (Proteintech, # 60097-1-Ig), Flag (Proteintech, # 20543-1-AP), HA (Proteintech, # 66006-2-Ig), His (Proteintech, # 66005-1-Ig), ubiquitin (CST, # 91112 S), K48 (CST, # 12805), K63 (CST, # 5621S). Secondary anti-mouse (# SA00001-1), and anti-rabbit (# SA00001-2) horseradish-coupled antibodies were from Proteintech.

### Co-immunoprecipitation

A375 and 293 T cells were lysed in an IP binding buffer (BeaverBeads protein A Immunoprecipitation Kit, 22202-20). After centrifugation, the supernatants were collected and incubated with protein A/G magnetic beads and 5 mg antibodies for 4 h at 4 °C followed by washing three times with IP washing buffer. The components of immunoprecipitate were investigated by SDS-PAGE gels, then followed by transferring onto the PVDF membranes and analyzed by corresponding antibodies. In the experiment of IP ubiquitin, proteasome inhibitors (MG132- HY-13259, MCE; Epoxomicin- HY-13821, MCE) and deubiquitinase inhibitors (PR-619- HY-13814, MCE) were additional needed.

### Galectin-3-coated beads and in vitro bioassays

Dynabeads M-280 Streptavidin (11-205-D, Invitrogen) were incubated with saturating amounts of recombinant human gal-3 (10289-H08H1, sinobiological) overnight at 4 °C. Then, beads were washed three times with PBS (containing 2 mM EDTA and 0.05% Tween-20). Under adding lactose (Solarbio, L8911), LacNAc or PBS, different amounts of gal-3-coated beads were incubated with the cytokines (IFN-γ, 11725-HNAS, sinobiological; IL-12, 10052-H02H, sinobiological) for 1 h at room temperature on a shaker. After magnetic sorting of the beads, the cytokines present in the supernatants were measured by ELISA (IFN-γ-JL12152, JiangLai; IL-12- JL29081, JiangLai).

### Cone beam CT (CBCT) scanning and 3D imaging

Mice with subcutaneous melanoma were scanned by an X-RAD SmART device (Precision X-Ray, INC) at the Experimental Center of Lanzhou University before they were sacrificed. Firstly, CBCT Scout was chosen to acquire a rapid, low dose and resolution image, then a high-resolution CBCT was run. The DCMFiles were opened by Radiantviewer. And the software was used for 3D modeling of subcutaneous tumors in mice.

### Statistical analysis

Statistical analysis was performed by GraphPad Prism 8 software. Data are presented as the mean ± standard deviation for each sample. Student’s *t*-test was used to determine the statistical differences between the two groups. The data of integrated optical density was obtained by ImageJ software. *P* < 0.05 was considered statistically significant.

## Supplementary information

Supplementary information

Supplementary Table S1

Supplementary Table S2

Supplementary Table S3

## Data Availability

All relevant data can be obtained from the corresponding author upon request.

## References

[1] Arozarena I, Wellbrock C (2019). Phenotype plasticity as enabler of melanoma progression and therapy resistance. Nat Rev Cancer.

[2] Dammeijer F, van Gulijk M, Mulder EE, Lukkes M, Klaase L, van den Bosch T (2020). The PD-1/PD-L1-Checkpoint Restrains T cell Immunity in Tumor-Draining Lymph Nodes. Cancer Cell.

[3] Robert C, Ribas A, Schachter J, Arance A, Grob JJ, Mortier L (2019). Pembrolizumab versus ipilimumab in advanced melanoma (KEYNOTE-006): post-hoc 5-year results from an open-label, multicentre, randomised, controlled, phase 3 study. Lancet Oncol.

[4] Flavahan WA, Gaskell E, Bernstein BE (2017). Epigenetic plasticity and the hallmarks of cancer. Science.

[5] Guo Y, Song Y, Guo Z, Hu M, Liu B, Duan H (2018). Function of RAD6B and RNF8 in spermatogenesis. Cell Cycle.

[6] Kuang J, Li L, Guo L, Su Y, Wang Y, Xu Y (2016). RNF8 promotes epithelial-mesenchymal transition of breast cancer cells. J Exp Clin Cancer Res.

[7] Li L, Guturi KKN, Gautreau B, Patel PS, Saad A, Morii M (2018). Ubiquitin ligase RNF8 suppresses Notch signaling to regulate mammary development and tumorigenesis. J Clin Investig.

[8] Lee HJ, Li CF, Ruan D, Powers S, Thompson PA, Frohman MA (2016). The DNA damage transducer RNF8 facilitates cancer chemoresistance and progression through twist activation. Mol Cell.

[9] Nowsheen S, Aziz K, Aziz A, Deng M, Qin B, Luo K (2018). L3MBTL2 orchestrates ubiquitin signalling by dictating the sequential recruitment of RNF8 and RNF168 after DNA damage. Nat Cell Biol.

[10] Huen MS, Grant R, Manke I, Minn K, Yu X, Yaffe MB (2007). RNF8 transduces the DNA-damage signal via histone ubiquitylation and checkpoint protein assembly. Cell.

[11] Costa Svedman F, Das I, Tuominen R, Darai Ramqvist E, Höiom V, Egyhazi Brage S (2022). Proliferation and immune response gene signatures associated with clinical outcome to immunotherapy and targeted therapy in metastatic cutaneous malignant melanoma. Cancers (Basel).

[12] Xu Y, Hu Y, Xu T, Yan K, Zhang T, Li Q (2021). RNF8-mediated regulation of Akt promotes lung cancer cell survival and resistance to DNA damage. Cell Rep.

[13] Wang L, Yang L, Wang C, Zhao W, Ju Z, Zhang W (2020). Inhibition of the ATM/Chk2 axis promotes cGAS/STING signaling in ARID1A-deficient tumors. J Clin Invest.

[14] Ouyang S, Song Y, Tian Y, Chen Y, Yu X, Wang D (2015). RNF8 deficiency results in neurodegeneration in mice. Neurobiol Aging.

[15] Perez-Guijarro E, Yang HH, Araya RE, El Meskini R, Michael HT, Vodnala SK (2020). Multimodel preclinical platform predicts clinical response of melanoma to immunotherapy. Nat Med.

[16] Greten FR, Grivennikov SI (2019). Inflammation and cancer: triggers, mechanisms, and consequences. Immunity.

[17] Ozga AJ, Chow MT, Luster AD (2021). Chemokines and the immune response to cancer. Immunity.

[18] Tait Wojno ED, Hunter CA, Stumhofer JS (2019). The immunobiology of the interleukin-12 family: room for discovery. Immunity.

[19] Kaczanowska S, Beury DW, Gopalan V, Tycko AK, Qin H, Clements ME (2021). Genetically engineered myeloid cells rebalance the core immune suppression program in metastasis. Cell.

[20] Garris CS, Arlauckas SP, Kohler RH, Trefny MP, Garren S, Piot C (2018). Successful anti-PD-1 cancer immunotherapy requires T cell-dendritic cell crosstalk involving the cytokines IFN-γ and IL-12. Immunity.

[21] Gollob JA, Mier JW, Veenstra K, McDermott DF, Clancy D, Clancy M (2000). Phase I trial of twice-weekly intravenous interleukin 12 in patients with metastatic renal cell cancer or malignant melanoma: ability to maintain IFN-gamma induction is associated with clinical response. Clin Cancer Res.

[22] Lei X, Lei Y, Li J-K, Du W-X, Li R-G, Yang J (2020). Immune cells within the tumor microenvironment: Biological functions and roles in cancer immunotherapy. Cancer Lett.

[23] Petitprez F, Meylan M, de Reyniès A, Sautès-Fridman C, Fridman WH (2020). The tumor microenvironment in the response to immune checkpoint blockade therapies. Front Immunol.

[24] Dai E, Zhu Z, Wahed S, Qu Z, Storkus WJ, Guo ZS (2021). Epigenetic modulation of antitumor immunity for improved cancer immunotherapy. Mol Cancer.

[25] Kim SJ, Kang HG, Kim K, Kim H, Zetterberg F, Park YS (2021). Crosstalk between WNT and STAT3 is mediated by galectin-3 in tumor progression. Gastric Cancer.

[26] Song L, Tang JW, Owusu L, Sun MZ, Wu J, Zhang J (2014). Galectin-3 in cancer. Clin Chim Acta.

[27] Vuong L, Kouverianou E, Rooney CM, McHugh BJ, Howie SEM, Gregory CD (2019). An orally active galectin-3 antagonist inhibits lung adenocarcinoma growth and augments response to PD-L1 blockade. Cancer Res.

[28] Gilson RC, Gunasinghe SD, Johannes L, Gaus K (2019). Galectin-3 modulation of T-cell activation: mechanisms of membrane remodelling. Prog Lipid Res.

[29] Kouo T, Huang L, Pucsek AB, Cao M, Solt S, Armstrong T (2015). Galectin-3 shapes antitumor immune responses by suppressing CD8+ T cells via LAG-3 and inhibiting expansion of plasmacytoid dendritic cells. Cancer Immunol Res.

[30] Vasiukov G, Novitskaya T, Zijlstra A, Owens P, Ye F, Zhao Z (2020). Myeloid cell-derived TGFβ signaling regulates ECM deposition in mammary carcinoma via adenosine-dependent mechanisms. Cancer Res.

[31] Demotte N, Bigirimana R, Wieers G, Stroobant V, Squifflet JL, Carrasco J (2014). A short treatment with galactomannan GM-CT-01 corrects the functions of freshly isolated human tumor-infiltrating lymphocytes. Clin. Cancer Res.

[32] Farhad M, Rolig AS, Redmond WL (2018). The role of Galectin-3 in modulating tumor growth and immunosuppression within the tumor microenvironment. Oncoimmunology.

[33] Hwang MP, Fecek RJ, Qin T, Storkus WJ, Wang Y (2020). Single injection of IL-12 coacervate as an effective therapy against B16-F10 melanoma in mice. J Controlled Release.

[34] Hicks KC, Chariou PL, Ozawa Y, Minnar CM, Knudson KM, Meyer TJ (2021). Tumour-targeted interleukin-12 and entinostat combination therapy improves cancer survival by reprogramming the tumour immune cell landscape. Nat Commun.

[35] Smith LK, Boukhaled GM, Condotta SA, Mazouz S, Guthmiller JJ, Vijay R (2018). Interleukin-10 directly inhibits CD8(+) T cell function by enhancing N-glycan branching to decrease antigen sensitivity. Immunity.

[36] Gordon-Alonso M, Hirsch T, Wildmann C, van der Bruggen P (2017). Galectin-3 captures interferon-gamma in the tumor matrix reducing chemokine gradient production and T-cell tumor infiltration. Nat Commun.

[37] Bao Y, Wang Z, Liu B, Lu X, Xiong Y, Shi J (2019). A feed-forward loop between nuclear translocation of CXCR4 and HIF-1α promotes renal cell carcinoma metastasis. Oncogene.

[38] Wang L, Li YS, Yu LG, Zhang XK, Zhao L, Gong FL (2020). Galectin-3 expression and secretion by tumor-associated macrophages in hypoxia promotes breast cancer progression. Biochem. Pharmacol.

[39] Fischöder T, Laaf D, Dey C, Elling L (2017). Enzymatic synthesis of N-acetyllactosamine (LacNAc) type 1 oligomers and characterization as multivalent galectin ligands. Molecules.

[40] Bumba L, Laaf D, Spiwok V, Elling L, Křen V, Bojarová P (2018). Poly-N-acetyllactosamine neo-glycoproteins as nanomolar ligands of human galectin-3: binding kinetics and modeling. Int J Mol Sci.

[41] Kubli SP, Berger T, Araujo DV, Siu LL, Mak TW (2021). Beyond immune checkpoint blockade: emerging immunological strategies. Nat Rev Drug Discov.

[42] Young A, Mittal D, Stagg J, Smyth MJ (2014). Targeting cancer-derived adenosine: new therapeutic approaches. Cancer Discov.

[43] Pitt JM, Marabelle A, Eggermont A, Soria JC, Kroemer G, Zitvogel L (2016). Targeting the tumor microenvironment: removing obstruction to anticancer immune responses and immunotherapy. Ann Oncol.

[44] Riera-Domingo C, Audige A, Granja S, Cheng WC, Ho PC, Baltazar F (2020). Immunity, hypoxia, and metabolism-the menage a trois of cancer: implications for immunotherapy. Physiol Rev.

[45] Gupta R, Somyajit K, Narita T, Maskey E, Stanlie A, Kremer M (2018). DNA repair network analysis reveals shieldin as a key regulator of NHEJ and PARP inhibitor sensitivity. Cell.

[46] Guo Z, Tian Y, Guo Y, Li B, Liu X, Xie K (2019). RAD6B plays a critical role in neuronal DNA damage response to resist neurodegeneration. Front Cell Neurosci.

