# Supplementary material for: RNF8 enhances the sensitivity of PD-L1 inhibitor against melanoma through ubiquitination of galectin-3 in stroma
Source: Cell Death Discov. 2023 Jun 30;9:205. doi: 10.1038/s41420-023-01500-3 (PMC10313721; doi:10.1038/s41420-023-01500-3)
Supplement: Supplementary file 1 — Supplementary information [file 41420_2023_1500_MOESM1_ESM.docx]

**Supplementary Information**

**RNF8 enhances the sensitivity of PD-L1 inhibitor aganst melanoma through ubiquitination of galectin-3 in stroma**

Yanan Guo ^1+^, Rong Shen ^1+^, Keren Yang ^1^, Yutong Wang^1^, Haoyun Song^1^, Xiangwen Liu ^1^, Xin Cheng ^1^, Rile Wu^1^, Yanfeng Song ^1^, Degui Wang ^1 2 *^ [^ORCID^](https://orcid.org/0000-0001-9923-2060)

^1^ School of basic medical sciences, Lanzhou University, Gansu, Lanzhou, 73000, China

^2^ NHC Key Laboratory of diagnosis and therapy of Gastrointestinal Tumor, Lanzhou, 730000, China

^+^ Contributed equally

^*^ Corresponding author

Yanan Guo: [guoyn2021@lzu.edu.cn](mailto:guoyn2021@lzu.edu.cn)

Rong Shen: [shenr@lzu.edu.cn](mailto:shenr@lzu.edu.cn)

Keren Yang: [yangkr18@lzu.edu.cn](mailto:yangkr18@lzu.edu.cn)

Yutong Wang: [ytwang2020@lzu.edu.cn](mailto:yangkr18@lzu.edu.cn)

Haoyun Song: songhy20@lzu.edu.cn

Xiangwen Liu: [liuxiangwen@lzu.edu.cn](mailto:liuxiangwen@lzu.edu.cn)

Xin Cheng: [chengx17@lzu.edu.cn](mailto:chengx17@lzu.edu.cn)

Rile Wu: [wurl20@lzu.edu.cn](mailto:wurl20@lzu.edu.cn)

Yanfeng Song: [songyanfeng@lzu.edu.cn](mailto:songyanfeng@lzu.edu.cn)

*Degui Wang: [wangdegui@lzu.edu.cn](mailto:wangdegui@lzu.edu.cn)


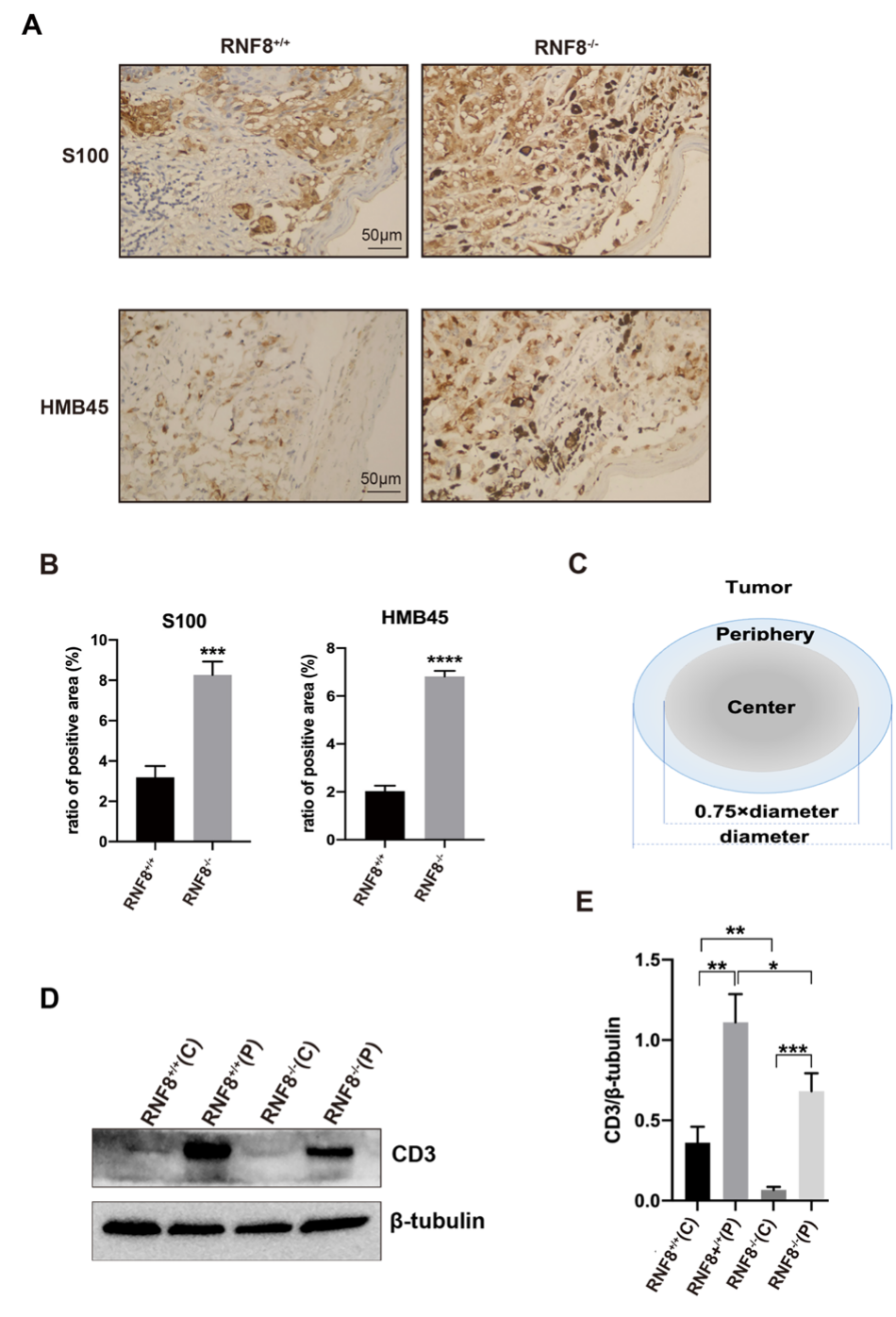


**Supplementary Fig. 1 RNF8 deficiency in host promoted implanted melanoma progression.** (A) Immunohistochemical staining of S100 and HMB45 in tumor-beared RNF8^+/+^ and RNF8^-/-^ mice. (B) Statistic analysis of S100 and HMB45 in (A). (C) The schematic diagram of tumor center (center, C) and periphery (periphery, P). (D) Immunoblot analysis of gal-3 in central and peripheral tumor in RNF8^+/+^ and RNF8^-/-^ mice. (E) Statistic analysis of CD3 in (D). Data are expressed as the mean ± SD (n = 3). Student's *t*-test; *P < 0.05, ***P* < 0.01, ****P* < 0.001, *****P* < 0.0001.


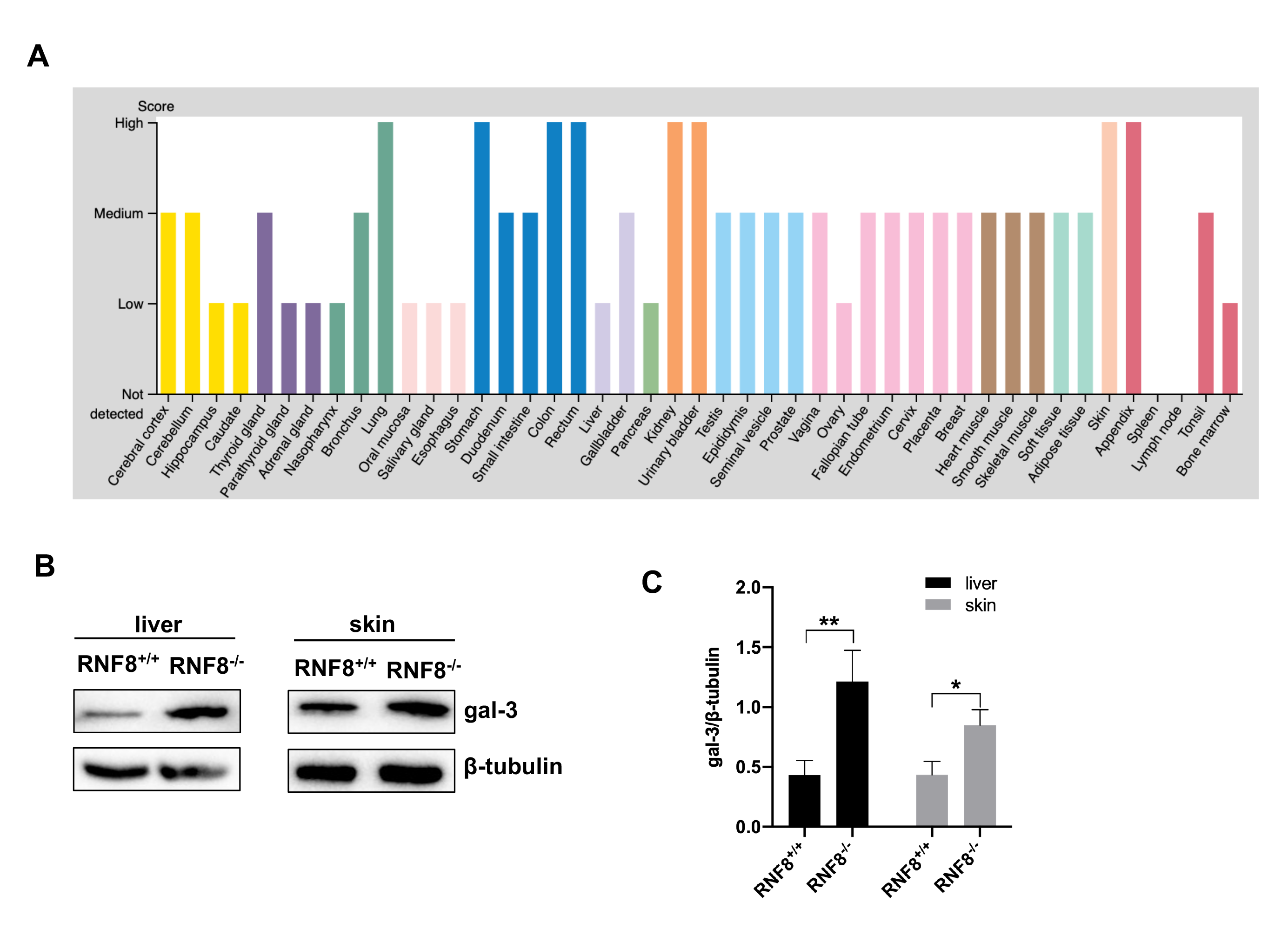


**Supplementary Fig. 2 The galectin-3 expression in mice. (**A) Galectin-3 protein level in different tissue from <https://www.proteinatlas.org>. (B-C) Western blot investigation of galectin-3 expression in liver and skin from RNF8^+/+^ or RNF8^-/-^ mice. Data are expressed as the mean ± SD (n = 3). Student's *t*-test; *P < 0.05, ***P* < 0.01.


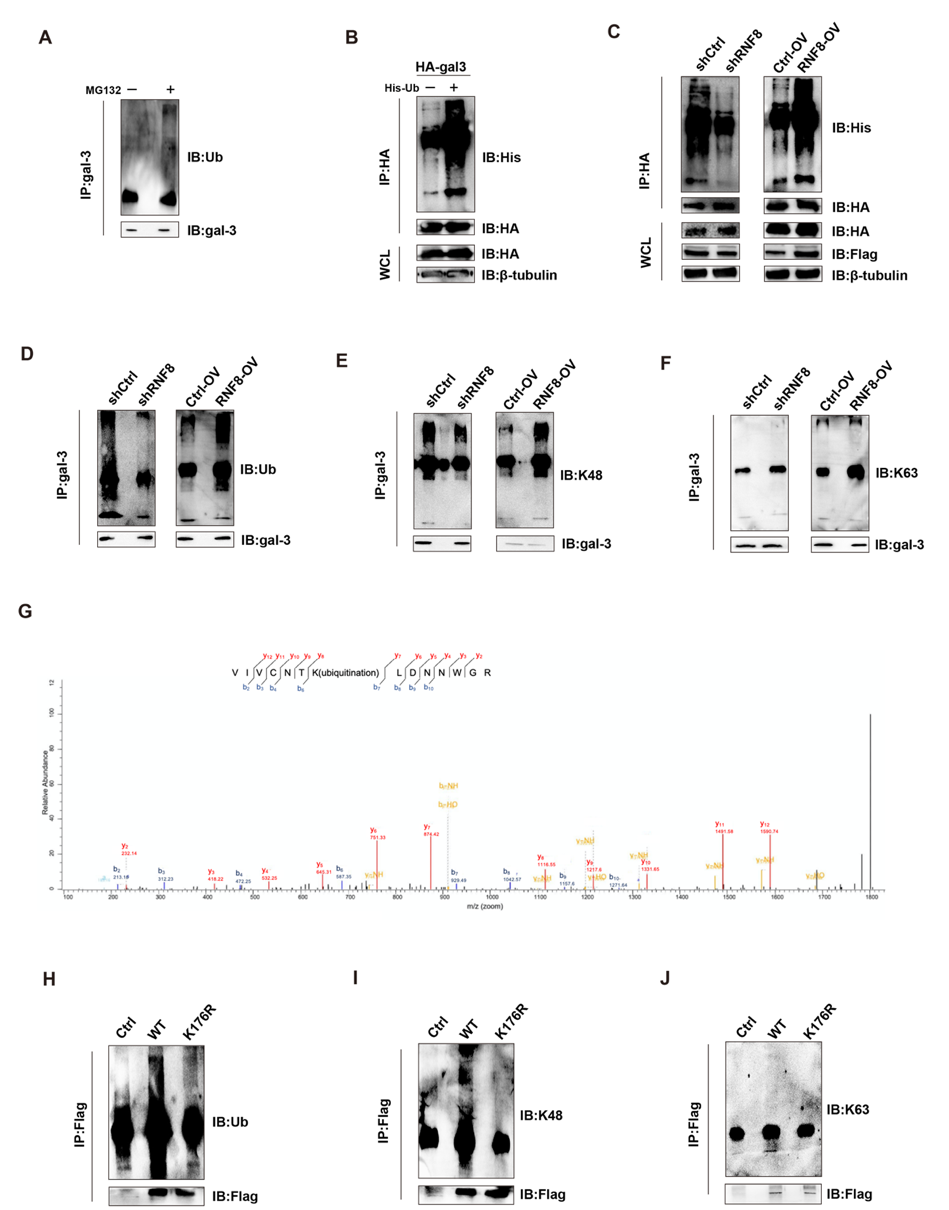


**Supplementary Fig.** **3 RNF8 mediated K48-linked polyubiquitination of gal-3.** (A) A375 cells were treated with or without 10 mmol/L MG132 for 4 h. The ubiquitination level of gal-3 was detected. (B) HA-tagged gal-3 and Ub plasmids were co-transfected into A375 cells, then IP and western blot was detected. (C) A375 cells were transfected with LV-RNF8 (shRNF8 or RNF8-OV), His-Ub was investigated. (D-F) The total ubiquitination (D), K48-linked (E) and K63-linked ubiquitination (F) of gal-3 in A375 cells. (G**)** The specific ubiquitination modification site of gal-3 was detected by LC-MS/MS. (H-J**)** A375 cells were transfected with Flag-Ctrl, Flag-LGALS3 and Flag-K176R, the ubiquitination (H) K48-linked (I) and K63-linked (J) ubiquitination were detected.


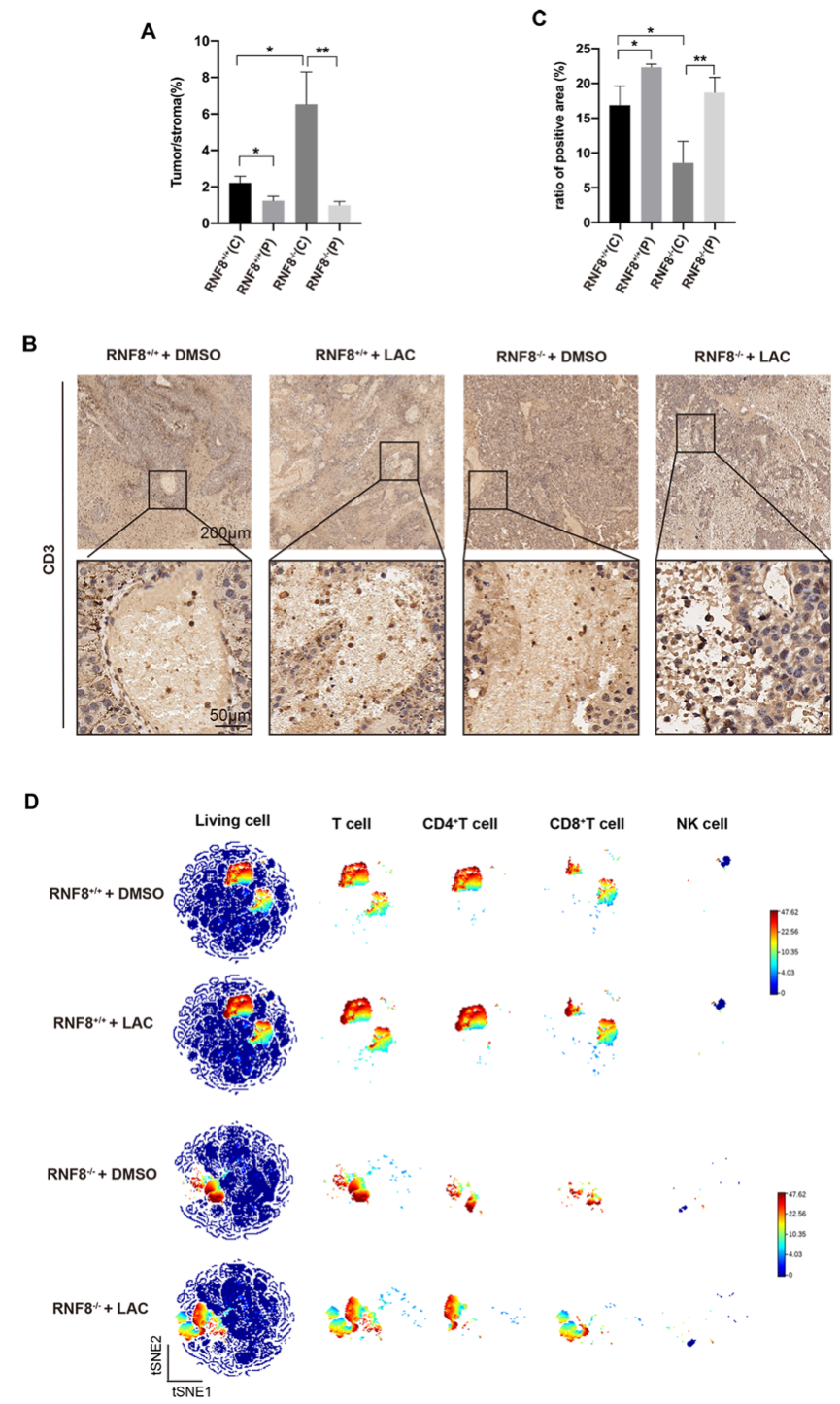


**Supplementary Fig.** **4 Inhibition of gal-3 increased the TILs in TME.** (A) The ratio of tumor area compared to stroma area in each group of Fig. 6C. （B） Immunohistochemical staining of CD3 in tumor-beared RNF8^+/+^ and RNF8^-/-^ mice with LAC or DMSO. (C) Statistic analysis of CD3 in (B), the values were presented as the mean ± SD (n = 3). Student's *t*-test; **P* < 0.05, ***P* < 0.01. (D) t-SNE plot of TILs in melanoma treated with or without LAC in RNF8^+/+^ and RNF8^-/-^ mice. including T cell, CD4^+^ T cell, CD8^+^ T cell and NK cell.


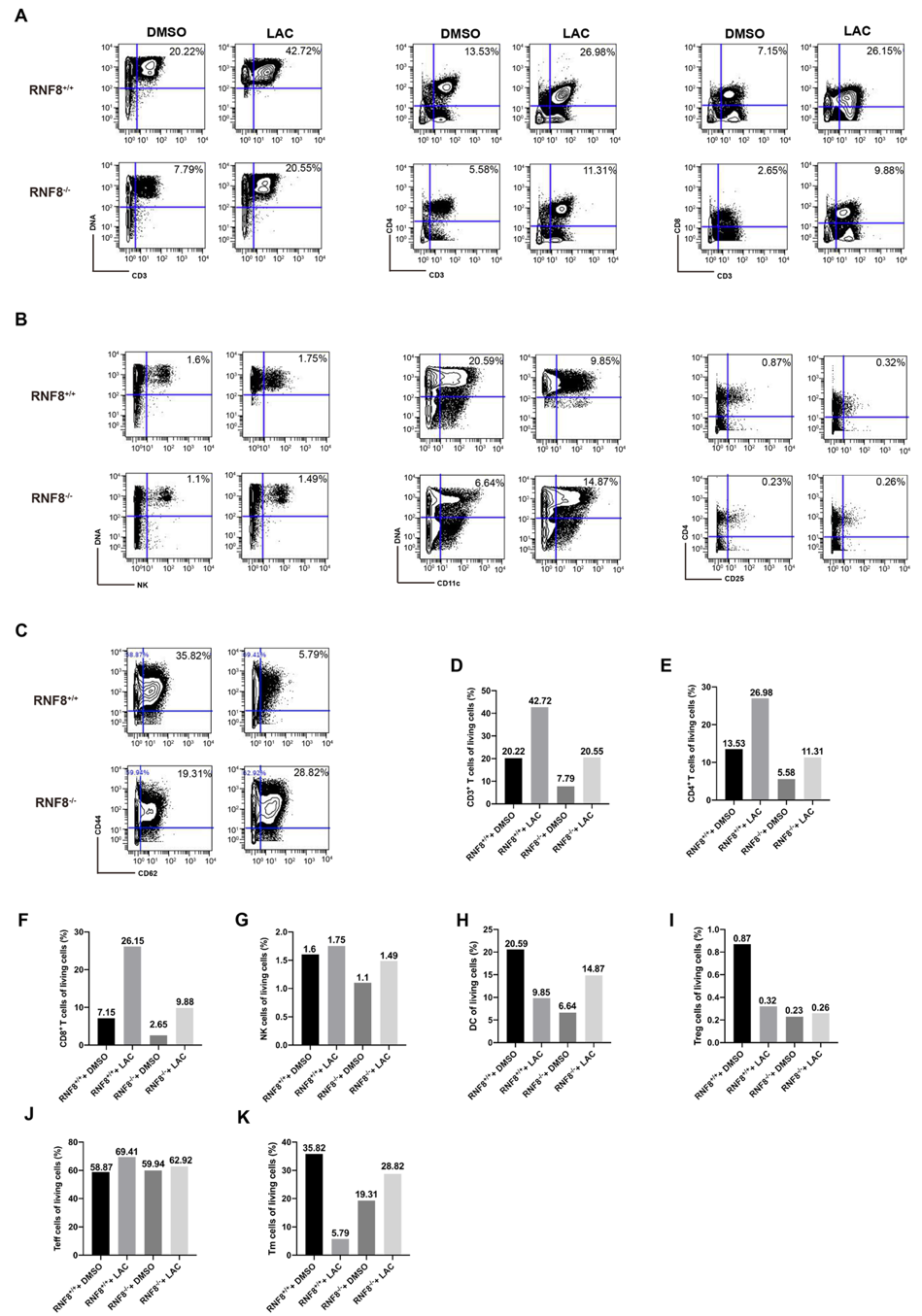


**Supplementary Fig. 5 Representative TILs subsets were changed** **after LAC intervention.** (A) The changes of CD4^+^ T cells (CD3^+^ CD4^+^) and CD8^+^ T cells (CD3^+^ CD8^+^). (B**)** The changes of NK cells, DC cells (CD11c) and regulatory T cells (Treg, CD4^+^ CD25^+^). (C**)** Changes of on effector T (Teff) and memory T (Tm) cells. (D-K**)** The Statistic analysis of corresponding markers in A-C, (D) CD3^+^ T, (E) CD4^+^ T, (F) CD8^+^ T, (G) NK, (H) DC, (I) Treg, (J) Teff and (K) Tm cells.


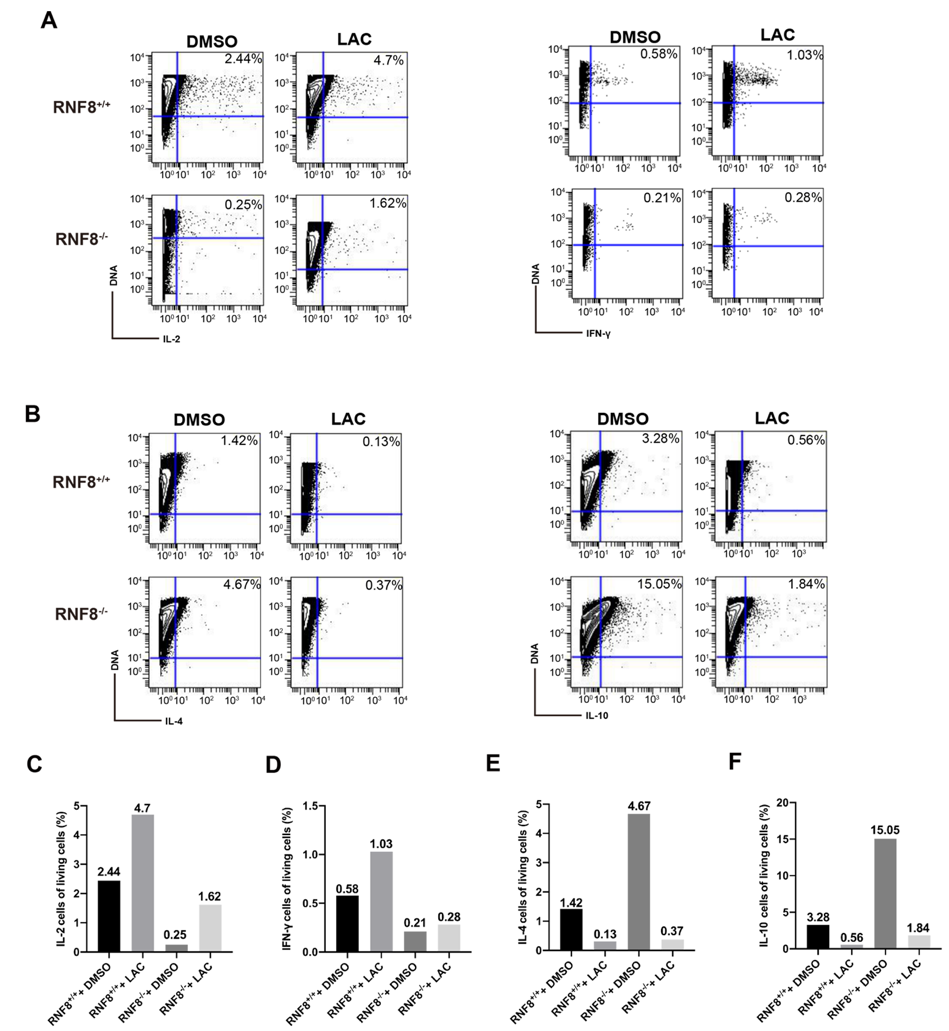


**Supplementary Fig. 6 Representative cytokines were changed after LAC intervention.** (A-B) The changes of cytokines IL-2, IFN-γ, IL-4 and IL-10 expression. (C-F) The Statistic analysis of (C) IL-2, (D) IFN-γ, (E) IL-4 and (F) IL-10.


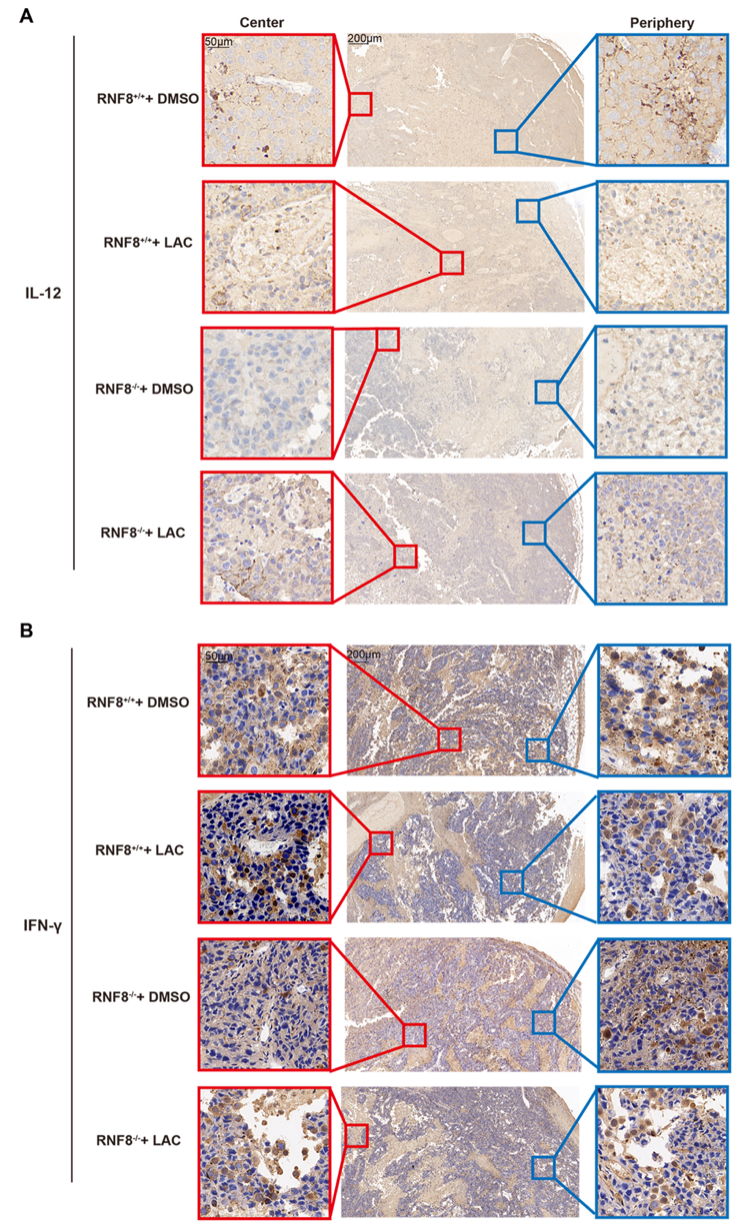


**Supplementary Fig. 7 Targeting gal-3 rescued the level of IL-12 and IFN-****γ in TME.** (A-B) Immunohistochemical staining of IL-12 (A) and IFN-γ (B) in tumor-beared RNF8^+/+^ and RNF8^-/-^ mice with LAC or DMSO. The tumor center was amplified in red box and tumor periphery was shown in blue box.


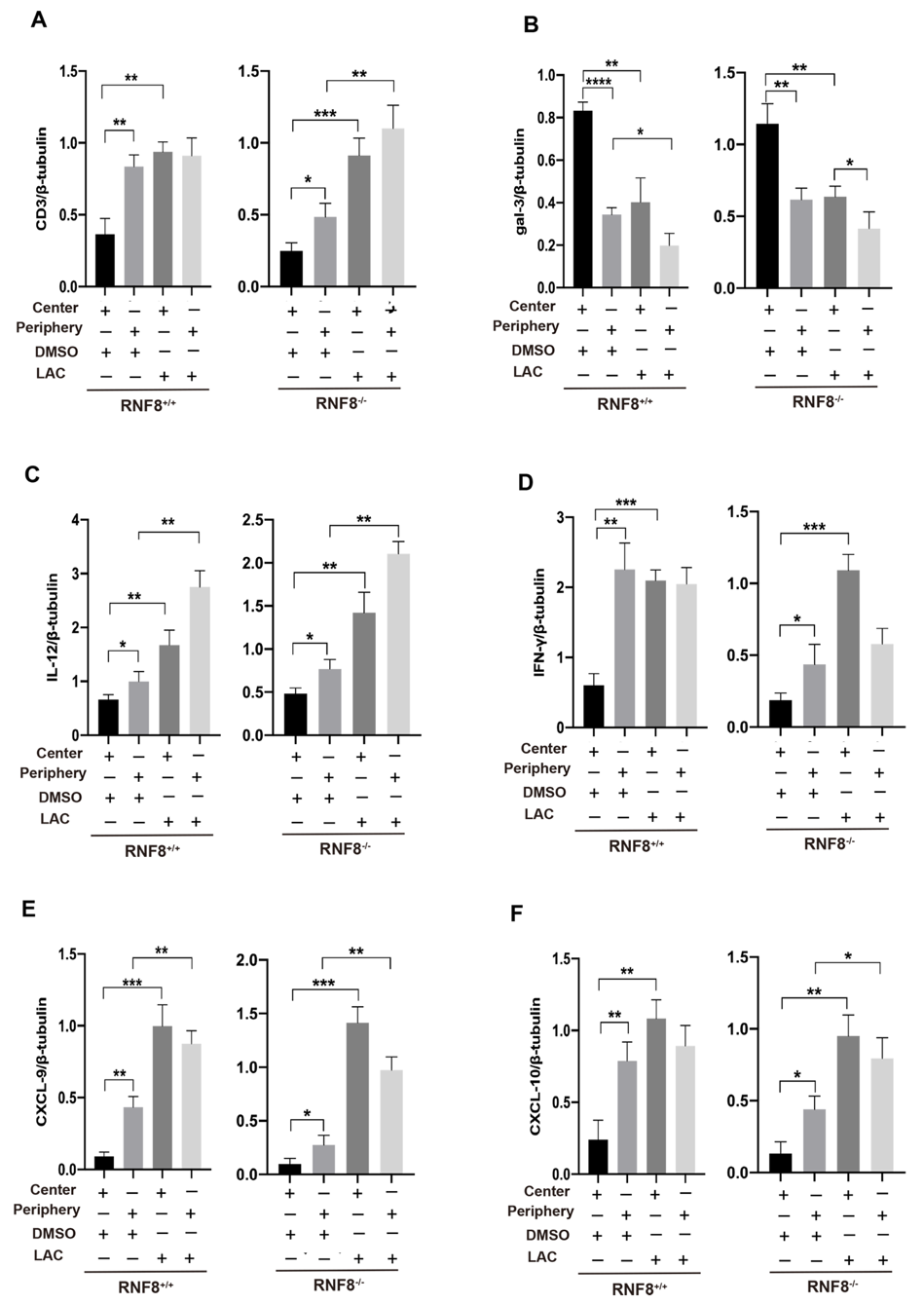


**Supplementary Fig. 8 Inhibition of gal-3 promoted the cytokines content in TME.** (A-F) The western blot analysis of CD3 (A), gal-3 (B), IL-12 (C), IFN-γ (D), CXCL-9 (E) and CXCL-10 (F) in central and peripheral tumor in Fig. 6I. All values were presented as the mean ± SD (n = 3). Student's *t*-test; **P* < 0.05, ***P* < 0.01, ****P* < 0.001, *****P* < 0.0001.


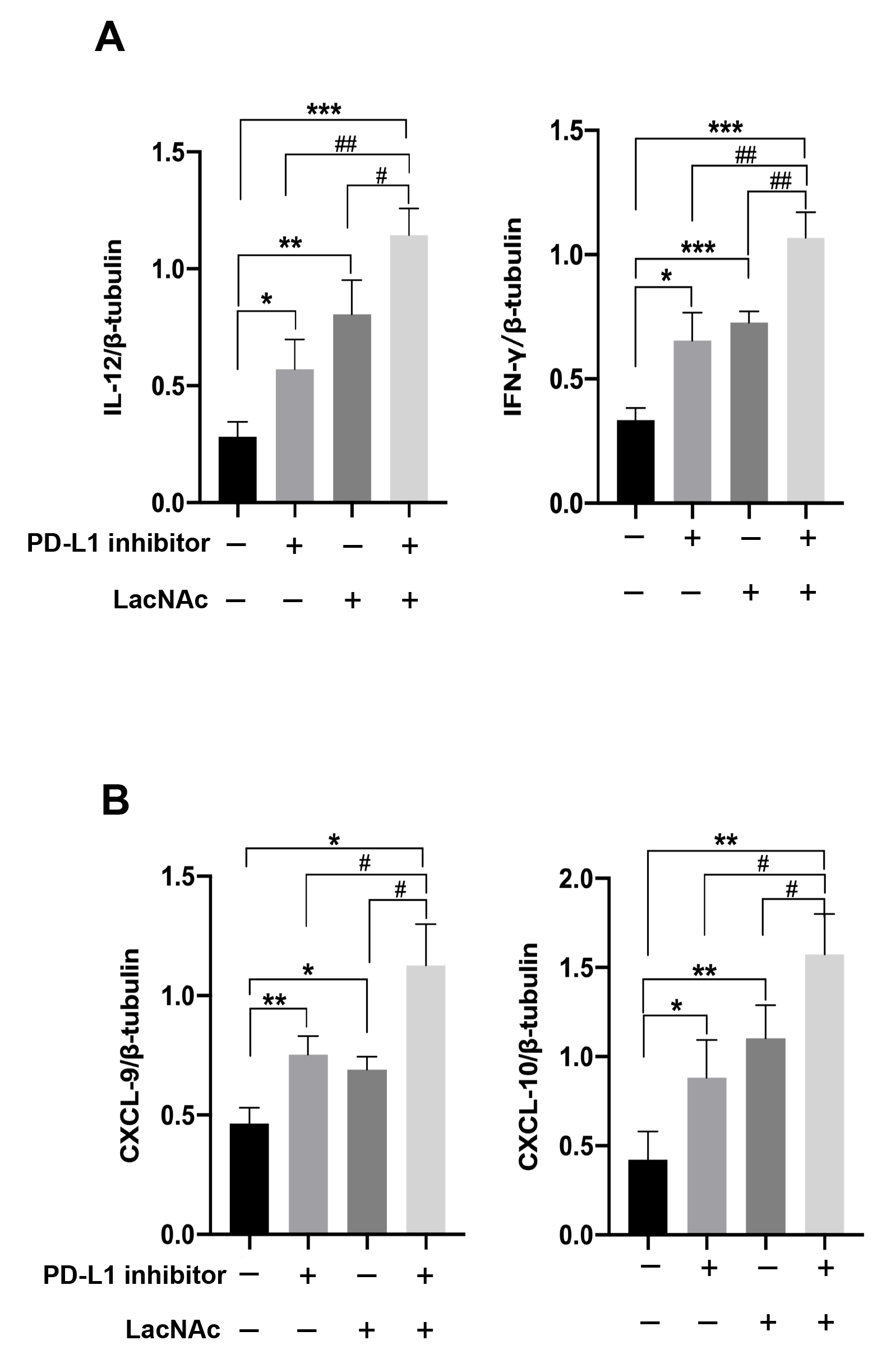


**Supplementary Fig. 9 Combination of PD-L1 inhibitor targeting gal-3 promoted the cytokines in TME.** (A-B) The western blot analysis of IL-12 and IFN-γ (A), CXCL-9 and CXCL-10 (B) in PD-L1 inhibitor, LacNAc and combination group in Fig. 7I. All values were presented as the mean ± SD (n = 3). Student's *t*-test; **P* < 0.05, ***P* < 0.01, ****P* < 0.001, *****P* < 0.0001.
