# Supplementary material for: RNF8 enhances the sensitivity of PD-L1 inhibitor against melanoma through ubiquitination of galectin-3 in stroma
Source: Cell Death Discov. 2023 Jun 30;9:205. doi: 10.1038/s41420-023-01500-3 (PMC10313721; doi:10.1038/s41420-023-01500-3)
Supplement: Supplementary file 2 — Supplementary Table S1 [file 41420_2023_1500_MOESM2_ESM.docx]

**Table S1. The sequencing of shRNF8**

| Primer | Primer sequences |
| --- | --- |
| RNF8-RNAi | TGCGGAGTATGAATATGAA |
