# Supplementary material for: RNF8 enhances the sensitivity of PD-L1 inhibitor against melanoma through ubiquitination of galectin-3 in stroma
Source: Cell Death Discov. 2023 Jun 30;9:205. doi: 10.1038/s41420-023-01500-3 (PMC10313721; doi:10.1038/s41420-023-01500-3)
Supplement: Supplementary file 3 — Supplementary Table S2 [file 41420_2023_1500_MOESM3_ESM.docx]

**Table S2. Mouse immune cell phenotyping antibodies of CyTOF**

| Mass and Tag | Antibodies | Ab Clone | Company |
| --- | --- | --- | --- |
| 163Dy | CD3ε | 145-2C11 | Biolegend |
| 159Tb  172Yb  153Eu | CD4  CD8a  CD335 | RM4-4  53-6.7  29A1.4 | Biolegend  Biolegend  Biolegend |
