# Supplementary material for: RNF8 enhances the sensitivity of PD-L1 inhibitor against melanoma through ubiquitination of galectin-3 in stroma
Source: Cell Death Discov. 2023 Jun 30;9:205. doi: 10.1038/s41420-023-01500-3 (PMC10313721; doi:10.1038/s41420-023-01500-3)
Supplement: Supplementary file 4 — Supplementary Table S3 [file 41420_2023_1500_MOESM4_ESM.docx]

**Table S3.** **Mouse intracellular cytokine antibodies of CyTOF**

| Mass and Tag | Antibodies | Ab Clone | Company |
| --- | --- | --- | --- |
| 165Hb | IFN-γ | XMG1.2 | Biolegend |
| 144Nd  166Er  158Gd | IL-2  IL-4  IL-10 | JES6-5H4  11B11  JES5-16E3 | Biolegend  Biolegend  Biolegend |
